# “Cross-talk” between gut microbiome dysbiosis and osteoarthritis progression: a systematic review

**DOI:** 10.3389/fimmu.2023.1150572

**Published:** 2023-04-25

**Authors:** Su Liu, Guoqing Li, Huihui Xu, Qichang Wang, Yihao Wei, Qi Yang, Ao Xiong, Fei Yu, Jian Weng, Hui Zeng

**Affiliations:** ^1^ Department of Bone and Joint Surgery, Peking University Shenzhen Hospital, Shenzhen, China; ^2^ National and Local Joint Engineering Research Center of Orthopaedic Biomaterials, Peking University Shenzhen Hospital, Shenzhen, China; ^3^ Department of Ultrasonography, Peking University Shenzhen Hospital, Shenzhen, China

**Keywords:** gut microbiome, osteoarthritis, cartilage, inflammation, immune response

## Abstract

**Objectives:**

The aim of this systematic review was to summarize the available literature on gut microbiome (GMB) and osteoarthritis (OA), analyze the correlation between GMB and OA, and explore potential underlying mechanisms.

**Methods:**

A systematic search of the PubMed, Embase, Cochrane, and Web of Science with the keywords “Gut Microbiome” and “Osteoarthritis” was conducted to identify the human and animal studies exploring the association between GMB and OA. The retrieval time range was from the database inception to July 31, 2022. Studies reported the other arthritic diseases without OA, reviews, and studies focused on the microbiome in other parts of the body with OA, such as oral or skin, were excluded. The included studies were mainly reviewed for GMB composition, OA severity, inflammatory factors, and intestinal permeability.

**Results:**

There were 31 studies published met the inclusion criteria and were analyzed, including 10 human studies and 21 animal studies. Human and animal studies have reached a consistent conclusion that GMB dysbiosis could aggravate OA. In addition, several studies have found that alterations of GMB composition can increase intestinal permeability and serum levels of inflammatory factors, while regulating GMB can alleviate the changes. Owing to the susceptibility of GMB to internal and external environments, genetics, and geography, the included studies were not consistent in GMB composition analysis.

**Conclusion:**

There is a lack of high-quality studies evaluating the effects of GMB on OA. Available evidence indicated that GMB dysbiosis aggravated OA through activating the immune response and subsequent induction of inflammation. Future studies should focus on more prospective, cohort studies combined with multi-omics to further clarify the correlation.

## 1 Introduction

Osteoarthritis (OA) is the most common musculoskeletal disease characterized by progressive articular cartilage loss, osteophyte formation, subchondral bone remodeling and joint inflammation (1, 2). OA is the main cause of joint pain and periarticular muscle weakness, followed by loss of function, increased disability, reduced performance of activities of daily living, and reduced health-related quality of life (3, 4). The aging global population and high incidence of obesity and joint injuries have increased the socioeconomic burden of OA, with an estimated 250 million people affected worldwide (1, 5, 6). Based on different exposure factors, OA is considered to be a collection of multiple phenotypes, each with specific pathophysiological and clinical features, such as metabolic OA, traumatic OA, and aging-related OA (7–9). These exposures alone or in concert contribute to the complex cross-talk between mechanical, biochemical, and cellular factors that ultimately lead to OA. Although OA is a typical joint degenerative disease, the role of inflammatory factors in its occurrence and development has attracted the attention of researchers. OA is characterized by chronic and low-grade inflammation, primarily mediated by the innate immune system (10, 11). As the pathogenesis of OA has not been completely elucidated, there is no recognized therapeutic target for advanced OA except pain management or joint replacement.

A previous large-scale study estimated that the human gut microbiome (GMB) contains approximately 35,000 bacterial species, of which 90% are members of the phyla *Firmicutes* and *Bacteroidetes* (12–14). GMB and its metabolites are closely related to host growth and development, immunity, and longevity (15), and perform a series of functions, such as maintaining metabolic homeostasis, developing and maturing the immune system, fighting infection, and producing neurotransmitters (16). Under physiological conditions, GMB can maintain a state of balance with the host to achieve symbiosis. However, this symbiosis can be disrupted and is termed GMB dysbiosis (17). Mancabelli et al. (18) conducted a meta-analysis of GMB in pre-agricultural and modern societies and showed that the modernization process altered the overall composition of human GMB by increasing and/or decreasing certain microbiota. According to previous studies, GMB has been closely associated with various pathological conditions, including obesity, diabetes, neurodegenerative diseases, cancer, and musculoskeletal diseases (19, 20). Based on the relationship between GMB and OA, experts have speculated that GMB dysbiosis may be closely related to the occurrence and development of OA (21–23); however, no consistent conclusion has been reached. In this systematic review, we aim to methodically summarize and analyze the latest evidence on GMB and OA in human and animal studies, systematically elaborate the “Gut-Joint” axis, and explore the underlying mechanisms by which GMB dysbiosis contributes to the progression of OA. With future research expected to deepen understanding of the correlation between GMB and OA, GMB dysbiosis may be a new target for the prevention or early treatment of OA.

## 2 Materials and methods

This systematic review of studies exploring the association between GMB and OA was conducted based on the method recommended by the Preferred Reporting Items for Systematic Review and Meta-Analyses (PRISMA) guidelines (2020) (Supplementary PRISMA 2020 Checklist) (24). It was registered on International Platform of Registered Systematic Review and Meta-analysis Protocols (INPLASY). The registration number is INPLASY202330039 and the protocol is available in full on inplasy.com (https://inplasy.com/inplasy-2023-3-0039/).

### 2.1 Search strategy and study selection

From database inception to July 31, 2022, we searched the PubMed, Embase, Cochrane, and Web of Science databases with the keywords “Gut Microbiome” and “Osteoarthritis” (Supplementary retrieval strategy). Inclusion criteria were human and animal studies exploring the association between GMB and OA. Exclusion criteria were arthritic diseases of other types, such as rheumatoid arthritis (RA), ankylosing spondylitis (AS); review; microbiome in other parts of the body with OA, such as oral or skin. Studies were screened by the order of duplication, title, abstract, and full text. In the process of analysis, if any literature was omitted, it was manually searched and included. The literature was screened by two independent researchers (Liu and Li), strictly adhering to uniform criteria. Any questions on selection of studies during the literature search were negotiated with each other or evaluated by a third party.

### 2.2 Study evaluation

Three reviewers (Liu, Li, and Xu) evaluated the studies independently. Following full text analysis, the selection of eligible studies was completed with the fourth reviewer (Wang). Any disagreements were discussed and reasons for inclusion or exclusion were shared.

### 2.3 Risk of bias evaluation

The quality of human studies was evaluated by Agency for Healthcare Research and Quality (AHRQ) scale on five aspects: selection bias, performance bias, attrition bias, detection bias, and reporting bias (25). The quality of animal studies was evaluated by the CAMARADES checklist on seven aspects: sample size calculation, random allocation, blinded evaluation of outcomes, appropriate animal model, animal welfare, peer review, and conflict of interest declaration (26). Three researchers (Liu, Li, Xu) evaluated the studies independently. Any questions were negotiated with each other or evaluated by a fourth researcher (Wang). The AHRQ evaluation scale scored study components as low risk, high risk, and unclear. Items from the CAMARADES checklist that were mentioned in a study were marked as “Yes”, otherwise were marked as “No”.

### 2.4 Data extraction and analysis

All data from the studies were extracted and evaluated in the same way. Extracted data included: author information, study design, evaluation methods, indicators correlated with GMB and OA, and the main findings. Considering that GMB dysbiosis could affect the intestinal mucosal barrier and inflammatory response, the indicators related to inflammation and intestinal permeability were also extracted.

## 3 Results

### 3.1 Study selection

**Figure 1:**
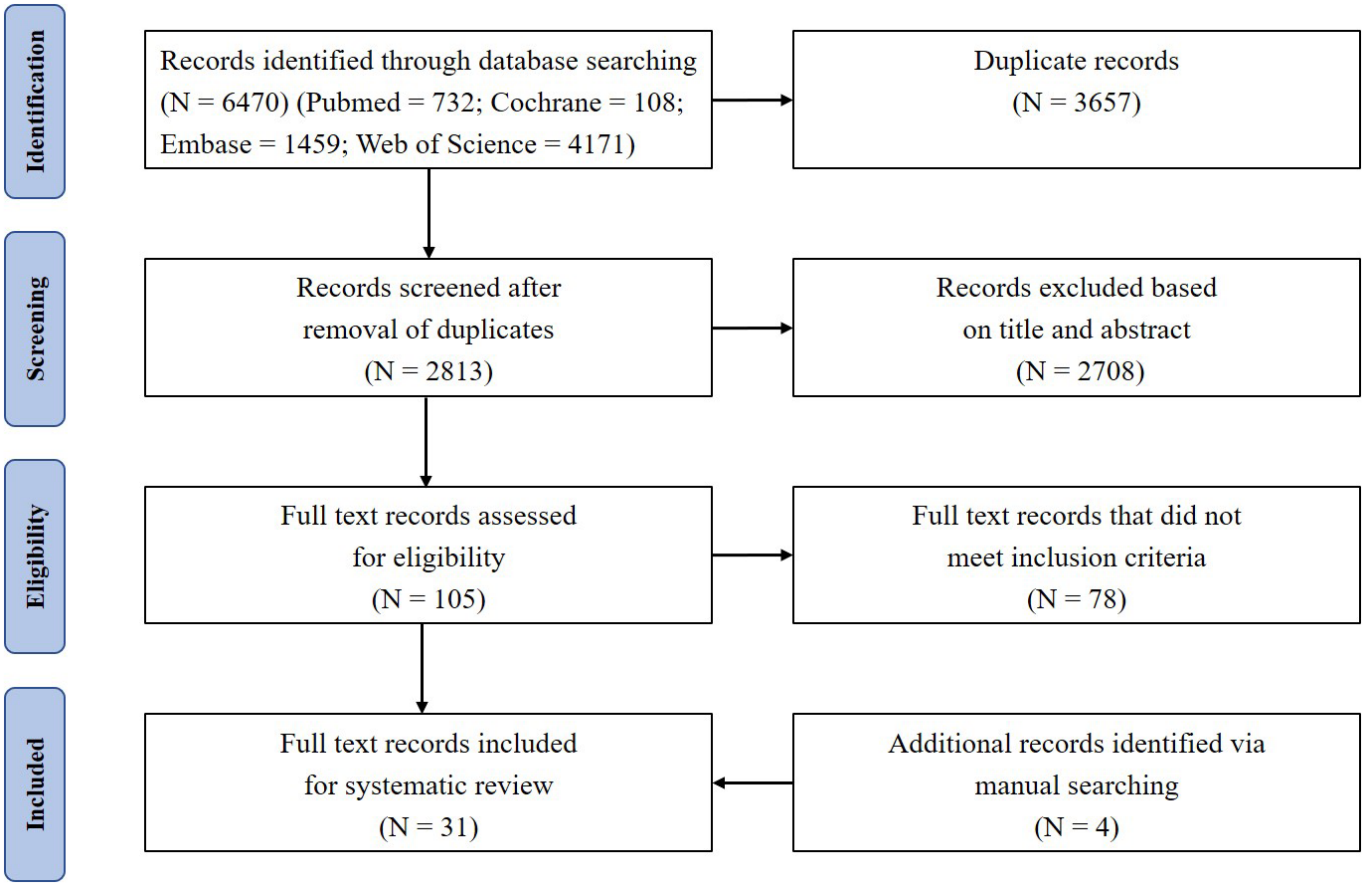
PRISMA flow diagram depicts the search results and selection of included studies in this systematic review.

A total of 6470 references were retrieved, including 732 in PubMed, 108 in Cochrane, 1459 in Embase, and 4171 in Web of Science. After sequential screening in order of duplication, title, abstract, and full text, 27 studies remained. The full-text assessment of excluded studies was shown in **Supplementary Table 1**. In the process of literature analysis, 4 studies were searched manually. Therefore, 31 studies were included in the final analysis—10 human studies (**Table 1**) and 21 animal studies (**Table 2**). The detailed literature retrieval process is shown in **Figure 1**.

**Table 1:** Human Studies on GMB and OA.

Study	Study Design	Sample	Country	Strict Inclusion or Exclusion Criteria	Sequencing Method	Correction for Confounding Factors and Method	Data analysis	Main Findings
27	Community-based observational study; N=1388	Stool	China	No	16S rRNA sequencing	Yes; MaAsLin	α-diversity; β-diversity; Relative abundance; Functional prediction	*Bilophila* and *Desulfovibrio* increased, *Roseburia* decreased; Aino acid, carbohydrate, and lipid metabolic changed in hand OA group.
28	Cross-sectional study; KOA with VDD (N=7); KOA (N=4); VDD (N=7); NC (N=6)	Stool	India	Yes	16S rRNA sequencing	No	α-diversity; β-diversity; Relative abundance; LEfSe	*Peptococcus*, *Shimwellia*, *Propionibacterium*, *Intestinimonas* and *Pavimonas* increased in KOA; *Parabacteroides*, *Butyricimonas* and *Gordonibacter* dominated in KOA with VDD; *Peptococcus*, *Intestimonas*, *Delftia* and *Oribacterium* dominated in KOA only.
29	Cross-sectional study; RA (N=9); OA (n=9)	Stool	Korea	Yes	16S rRNA sequencing	No	α-diversity; β-diversity; Relative abundance	*B/F* lower in RA; *Lactobacilli* and *Prevotella* enriched in RA; *Bacteroides* and *Bifidobacterium* enriched in OA.
30	Case-control study; OA (N=57,20 twins); Control (N=57, 2 twins)	Stool	United Kingdom	Yes	Metagenomic shotgun sequencing	Yes; Unpaired and paired Wilcoxon rank-sum test	α-diversity; β-diversity; Relative abundance; Functional prediction	Richness and diversity, *Bifidobacterium longum* and *Faecalibacterium prausnitzii*, energetic metabolism and acetate production decreased; *Clostridium spp.* increased in OA group.
31	Non-blinded randomized clinical trial; GLM (N=21); GS (N=17)	Stool	Australia	Yes	MALDI-TOF MS analysis	No	Relative abundance; WOMAC; Adverse events; Safety measures	*Clostridium* and *Staphylococcus* decreased, *Lactobacillus*, *Streptococcus* and *Eubacterium* increased in both groups; *Bifidobacterium* increased, *Enterococcus* and *yeast* deceased in GLM; *Bacteroides* decreased, *yeasts* and *Coliforms* increased in GS; OA improved in both groups.
32	Rotterdam Study (N=1427); Lifelines-DEEP Study (N=867)	Stool	Netherlands	No	16S rRNA sequencing	Yes; MaAsLin	α-diversity; β-diversity; Relative abundance; WOMAC	*Streptococcus* abundance associated with knee pain and joint inflammation.
33	Randomized, double-blind, placebo-controlled clinical trial; (N=80)	None	China	Yes	None	No	WOMAC; VAS; Serum CTX-II and CRP	TCI633 improved pain and stiffness of knee OA, as well as the serum CTX-II and CRP compared with placebo.
34	Randomized double-blind, placebo-controlled clinical trial; (N=433)	None	China	Yes	None	No	WOMAC; VAS; Serum CRP	*Lactobacillus casei Shirota* improved WOMAC and VAS scores, as well as the level of CRP; CRP strongly correlated with WOMAC and VAS.
35	Case-control Study; Over-weighted KOA (N=86); BMI matched subjects (N=96)	Stool	China	Yes	16S rRNA sequencing	No	Bacterial diversity and taxonomic; LEfSe; Random Forest model analysis	Diversity and richness decreased in over-weighted OA; 9 phyla and 87 genera different between two groups, dominated by *Gemmiger*, *Klebsiella*, *Akkermansia*, *Bacteroides*, *Prevotella*, *Aliistipes* and *Parabacteroides*.
36	Two sample MR	None	None	None	None	Yes; Comprehensively sensitive analyses; multivariable MR analyses; Reverse-direction MR analyses	Microbial taxa; GO Enrichment	*Methanobacteriaceae*, *Desulfovibrionales*, and *Ruminiclostridium5* negatively associated with knee OA; *RuminococcaceaeUCG003* and *Enterorhabdus* negatively associated with hip OA; 49 GO associated with knee OA; 3 GO associated with hip OA

GMB, gut microbiome; OA, osteoarthritis; N, number; NC, normal control; MaAsLin, Mltivariate linear regression analyses; KOA, knee osteoarthritis; VDD, vitamin D deficient; LEfSe, linear discriminant analysis effect size; GMB, gut microbiome; RA, rheumatoid arthritis; B/F, Bacteriodetes/Firmicutes; GLM, green-lipped mussel; GS, glucosamine sulphate; WOMAC, Western Ontario McMaster Universities Arthritis Index; VAS, visual analogue scale; CRP, C-reactive protein; TCI633, Streptococcus thermophilus; CTX-II, C-terminal telopeptide II; BMI, body mass index; MR, Mendelian Randomization; GO, Gene Ontology.

**Table 2:** Animal Experiments on GMB and OA.

Study		Study design	Animal Model	Intervention	Sample	Sequencing Method	Data analysis	Time points	Main findings
37		Female rhesus macaque: OA (N=10); NC (N=10)	Spontaneous OA	None	Stool	Metagenomics Sequencing	α-diversity; β-diversity; Functional prediction	None	*Mollicutes*, *Tenericutes*, *Coprobacillus* and *Faecalitalea* may be specific biomarkers for OA; *Lactobacillus* increased, *Prevotella* and *Ruminococcus* decreased in OA; Zinc/manganese transport system permease protein and Cyclopropane-fatty-acyl-phospholipid synthase enriched in OA.
38		3 male/3 female: Saline/MLI+;OA-METS-/MLI-; OAMETS-/MLI+; 6 male/6 female: OA+METS-/MLI; OA+METS+/MLI; SPF/MLI+	MLI	Fecal transplantation	Stool	16S rRNA sequencing	α-diversity; β-diversity; Relative abundance; Inflammatory factors; Gut permeability	8 weeks	GF mice with no fecal transplantation had the lowest histological OA severity; GF mice transplanted faeces from OA with METS had the highest inflammatory factors, gut permeability and OA severity; Increased *Fusobacterium* and *Faecalibaterium*, decreased *Ruminococcaceae* associated with OA severity and systemic inflammatory factors.
39		LFD or HFD supplemented with a control fiber or prebiotic (N=3/ time point)	DMM	LFD or HFD; Fiber or Prebiotic	Stool	16S rRNA sequencing	α-diversity; β-diversity; Relative abundance; Inflammatory factors; OARSI	12 weeks	*B*/*F* reduced in the obese mice, while partially corrected by oligofructose; Serum LPS and macrophage increased in the colon of obese mice, while reduced by oligofructose; IL-12 and MCP-1 reduced, IL-10 increased by oligofructose; oligofructose alleviated obesity-related cartilage degeneration.
40		TLR5KO + METS (N=11); TLR5KO + antibiotic (N=10); HFD (N=11); NC (N=11)	Cyclic compressive loading	TLR5KO and HFD	Stool	16S rRNA sequencing	OARSI; Inflammatory factors; α-diversity; β-diversity	2 weeks and 6 weeks	Severe obesity increased OARSI scoring; TLR5KO with antibiotic had the lowest OARSI scoring; GMB composition quite different among groups, especially in *Bacteroidetes* and *Firmicutes*.
41		DIO group (N=21); Chow group (N=11)	None	HFS	Stool	16S rRNA sequencing	Modified Mankin scoring; β-diversity	28 weeks	DIO group had higher modified Mankin scores and LPS; Body fat associated with modified Mankin score; *Lactobacillus species (spp.)* and *Methanobrevibacter spp.* strongly correlated with modified Mankin score.
21		GF male mice(13.5 weeks, N=15; 43 weeks, N=5); Strain matched SPF male mice(13.5 weeks, N=17; 43 weeks, N=6)	DMM	None	Stool	16S rRNA sequencing	ACS scoring; Osteophyte evaluation; LBP and LPS; α-diversity; β-diversity	8 weeks	ACS score decreased in GF mice, especially in young; Osteophyte size and LBP reduced in younger GF mice; Relative abundance of some OTUs differed between SPF mice with high and low ACS scores.
42		Sedentary + HFS; Aerobic exercise + HFS; Prebiotic fiber + HFS; Aerobic exercise + prebiotic fiber + HFS;(N=12/group)	Progressive moderate treadmill training	Prebiotic fiber and HFS	Cecal	16S rRNA sequencing	Inflammatory factors; Cecal microbiota; OARSI	12 weeks	Prebiotic fiber and aerobic exercise alleviated cartilage damage, prevented local and systemic leptin increasing, prevented insulin sensitivity increasing, prevented serum LPS increasing, improved serum lipid profile; Prebiotic fiber prevented the microbial dysbiosis.
43		*L. casei*; Gln; CII and Gln; L. casei, CII, and Gln; PBS; (N=8/group)	MIA	L. casei; CII; Gln	None	None	Pain behavior; OARSI; Inflammatory factors	8 weeks	*L. casei* combined with CII and Gln reduced pain, cartilage destruction, lymphocyte infiltration,pro-inflammatory cytokines and MMPs, while increased anti-inflammatory cytokines.
44		NC; IM; MIA; ID-CBT5101 (1 x 10^8^ CFU/day) and (1 x 10^10^ CFU/day); (N=10/group)	MIA	ID-CBT5101; IM	None	None	Cytokines; bone metabolism; Cartilage morphology	6 weeks	IDCBT5101 decreased inflammatory and bone metabolic markers, while increased IFN-γ and glycosaminoglycan; IDCBT5101 inhibited MMPs and TIMPs, preserved cartilage and synovium, reduced fibrous tissue.
45		Vehicle; Celecoxib; Probiotic complex + zinc + rosavin; (N=3/group)	MIA	Celecoxib; Probiotic complex, zinc, and rosavin	None	None	Pain behavior; Inflammatory factors; Microstructure of femurs	6 weeks	Combination group improved pain, prevented cartilage damage, decreased proinflammatory cytokines and catabolic factors, increased anti-inflammatory cytokines and anabolic factors.
46		Chow WT (N=22); Chow LD (N=15); HFD WT (N=17); HFD LD (N=8); MEF-R (N=15); WF-R (N=12)	DMM	HFD	Stool	16S rRNA sequencing	Modified Mankin Score; LPS; α-diversity; β-diversity; Relative abundance	12 weeks	LPS increased, *B*/*F* reduced in HFD; HFD strongly correlated with Modified Mankin Score; 9 genera significantly associated with Modified Mankin Score.
47		SD + sedentary; SD + exercise; HFD + sedentary; HFD + exercise; HFD + Berberine; (N=6/group)	None	HFD; Berberine	Stool	16S rRNA sequencing	Relative abundance; Mankin score; Thickness of cartilage layer; LPS	4 weeks	Diversity and protective bacteria decreased, LPS-producing bacteria, LPS, TLR4, MMP-13, and cartilage degeneration increased after HFD; Exercise alleviated the results; Berberine reduced LPS and GMB diversity.
48		NC-female; NC-OA-female; ABT-OA-female; NC-male; NC-OA-male; ABT-OA-male; (N=9/group)	DMM	Ampicillin; Neomycin	Stool	16S rRNA sequencing	OARSI; LPS, IL-6, TNF-α, calcium and magnesium ion; Bacteria in Phylum	8 weeks	ABT caused GMB dysbiosis; BV/TV, OASRI score, osteophyte score, osteophyte maturity, IL-6, TNF-α, calcium, and MMP-13 decreased by ABT, while magnesium and estrogen increased; Female and male mice respond differently to ABT.
49		WT + HFD; TLR5KO + chow; WT + chow; TLR5KO + antibiotics + chow; (N=11/group)	Cyclic compressive loading	Ampicillin; Neomycin	Stool	16S rRNA sequencing	OARSI; α-diversity; β-diversity; Bacterial community	6 weeks	GMB composition different among groups; Although high intensity circulatory loading resulted in cartilage loss, there was no significant difference among groups.
50		NC; HFD; HFD + CS; HFD + *M5*; HFD + CS-M5; (N=3/group)	HFD	*M5* and CS	Stool	16S rRNA sequencing	OARSI; CII; Leptin; α-diversity; β-diversity; Bacterial community	12 weeks	*M5* and CS protected cartilage and CII, reduced leptin; *Ruminococcaceae_UCG-014*, *Streptococcus* and *Bacteroides* strongly correlated with OA.
51		NC; MIA; Moxibustion; Diclofenac; (N=9/group)	MIA	Moxibustion; Diclofenac sodium	Stool	16S rRNA sequencing	Mankin score; Inflammatory Factors; α-diversity; β-diversity; Bacterial community	4 weeks	Cartilage damaged, IL-1β, TNF-α, and LPS increased, diversity decreased by MIA; Moxibustion improved the results; Moxibustion regulate GMB.
52		LR-2 (N=6); vehicle (N=6). Repeated 3 times.	MIA	LR-2	None	None	Pain Behavior; Mankin score; Intestinal structure and inflammation; LR-2 on chondrocytes	4 weeks	Pain and cytokine decreased, cartilage damage alleviated by LR-2; Intestinal damage and inflammation improved by LR-2; LR-2 improved inflammation in chondrocytes.
53		NC (N=9); ACLT (N=8); ACLT+TCI633 (5x10^11^CFU/kg/day, N=8; 5x10^10^ CFU/kg/day, N=9; 5x10^9^ CFU/kg/day, N=9); ACLT+GS (N=9).	ACLT	TCI633; GS	None	None	Pain behavior; Knee swelling; OARSI; Synovial inflammation; chondrocyte apoptosis	20 weeks	TCI633 and GS improved pain and knee swelling; TCI633 improved synovial inflammation, cartilage damage, and chondrocyte apoptosis in dose-dependent.
54		NC (16 weeks, N=5); NC (28 weeks, N=12); LIC (N=12); LIC + Vitamin C (N=12)	Spontaneous OA	LIC; Vitamin C	None	None	OARSI; Synovial inflammation; Inflammatory cytokine	Every 2 weeks	OARSI lower in LIC + Vitamin C than NC; Synovial inflammation no difference among groups; Inflammatory cytokine decreased by LIC.
55		Healthy dogs (N=13); AD dogs (N=14)	None	Semi-moist complete diet; Omega 3 fatty acids	Stool	16S rRNA sequencing	CRP, vitamin B12, folate; β-diversity; Bacterial community	0 and 45 days	CRP higher, while vitamin B12 and folate lower in AD group; *Megamonas* higher, while *Paraprevotellaceae*, *Porphyromonadaceae*, and *Mogibacteriaceae* lower in AD group.
56		Sedentary; Physically active; (N= 18/group)	Spontaneous OA	None	Stool	16S rRNA sequencing	Cartilage structure; OARSI; α-diversity; β-diversity	22 weeks	OA more severity in sedentary group; Aggrecan quantity lower in sedentary group; No difference in cartilage thickness, stiffness, and GMB composition between groups.

GMB, gut microbiome; OA, osteoarthritis; N, number; MLI, meniscal ligamentous injury; METS, metabolic syndrome; SPF, specific pathogen free; GF, germ free; DMM, destabilization of the medial meniscus; LFD, low fat diet; HFD, high fat diet; B/F, Bacteriodetes/Firmicutes; OARSI, Osteoarthritis Research Society International; LPS, lipopolysaccharide; IL-6, interleukin-6; MCP-1, monocyte chemoattractant protein-1; TLR5KO, Toll-like receptor-5 deficient; GMB, gut microbiome; DIO, diet-induced obese; ACS, articular cartilage structure; LBP, LPS binding protein; OTU, operational taxonomic units; HFS, high fat/high sucrose diet; L. casei, Lactobacillus case; Gln, glucosamine; CII, type II collagen; PBS, phosphate buffer saline; MIA, monosodium iodoacetate; MMP, matrix metalloproteinase; NC, normal control; IM, indomethacin; ID-CBT5101, Clostridium butyricum; CFU, colony forming units; TIMP, tissue inhibitors of metalloproteinases; WT, wild-type; LD, lipodystrophy; MEF-R, mouse embryonic fibroblast transplant; WF-R, wildtype fat transplant; SD, standard diet; TNF-α, tumor necrosis factor-α; Con, control; ABT, antibiotic treatment; BV, bone volume; TV, total volume; CS, chondroitin sulfate; M5, L. paracasei subsp. paracasei M5; LR-2, L. rhamnosus; ACLT, anterior cruciate ligament transection; TCI633, Streptococcus thermophilus; GS, glucosamine sulfate; LIC, lyophilized inactivated culture from Bifidobacterium longum CBi0703; AD, arthritis disease; CRP, C-reactive protein.

### 3.2 Basic characteristics of included studies

**Figure 2:**
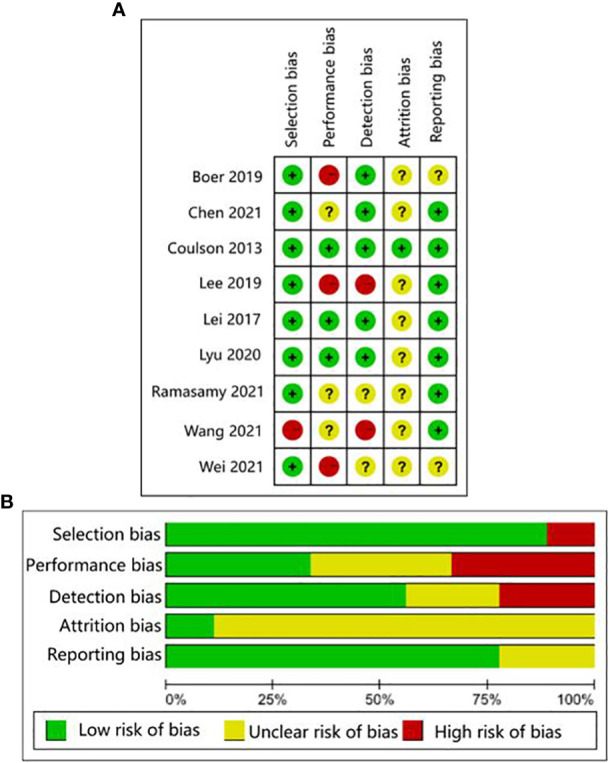
AHRQ evaluation for human studies. **(A)** Traffic-lights plots; **(B)** Weighted bar plots.

**Figure 3:**
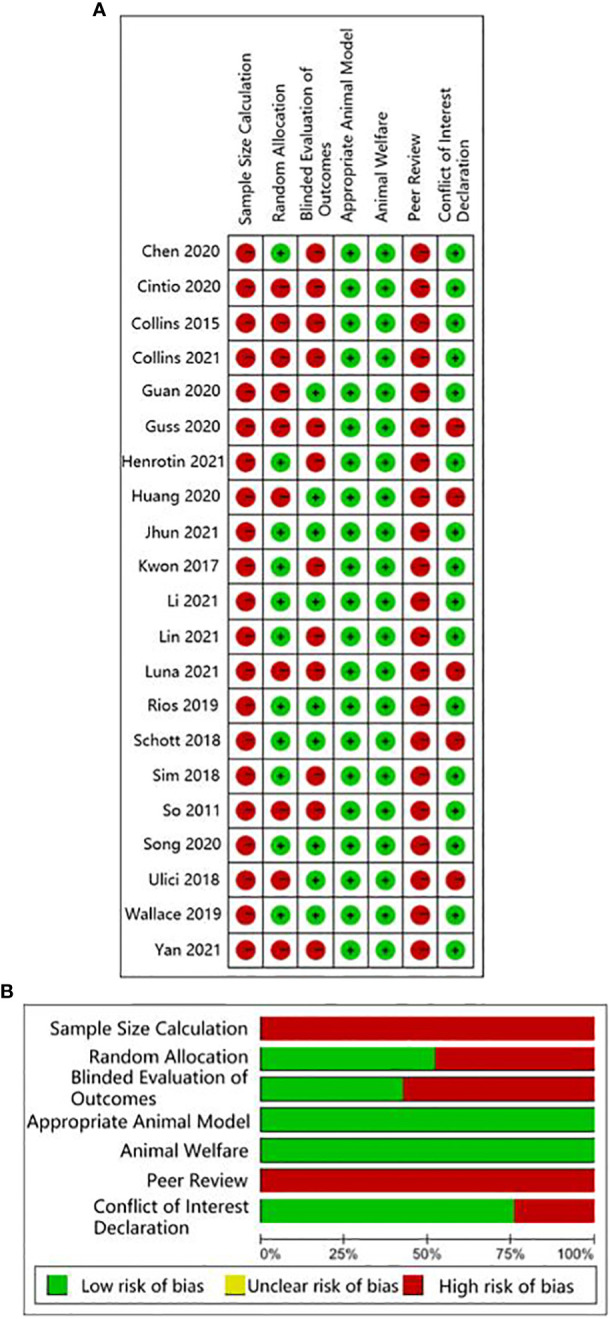
CAMARADES evaluation for animal experiments. **(A)** Traffic-lights plots; **(B)** Weighted bar plots.

The human studies comprised six observational studies (27–30, 32, 35), a two-sample Mendelian randomization (MR) study (36), and three randomized controlled trials (RCTs) (31, 33, 34). The observational studies were conducted to compare OA patients with normal controls or RA patients to identify differences in GMB composition and function. The MR study initially found the causal relationship between GMB and OA through large-scale genome-wide association studies (GWAS) summary statistics. The RCTs reported that supplemented with green-lipped mussels (GLM) (31), glucosamine sulfate (GS), *Streptococcus thermophilus* (TCI633) (33), and *Lactobacillus casei* Shirota (34) can significantly alleviate the symptoms of OA by regulating GMB. The basic characteristics of participants in human studies was shown in **Supplementary Table 2**. The animal studies included three non-intervention experiments (37, 55, 56), seven experiments about disturbing GMB to exacerbate OA (21, 38, 40, 41, 46, 48, 49), and 11 experiments about regulating GMB dysbiosis to alleviate OA (39, 42–44; 45, 47, 50–54). The risk of bias was evaluated for each study (**Figures 2**, **3**; **Supplementary Table 3**, **4**). Among the human studies, only one (31) had all five items as low risk, while the remaining eight studies had high risks and/or unclear, especially for attrition bias, which was not clearly indicated in the studies. The sample size and peer review declaration were not included in all of the animal studies. All of the animal experiments were conducted using currently recognized and established animal models, and the process was in line with animal ethics requirements.

### 3.3 Human studies: Observational studies reporting the association between GMB and OA

Wei et al. (27) found a significant correlation between β-diversity and symptomatic hand OA. Patients with symptomatic hand OA showed higher relative abundance of *Bilophila* and *Desulfovibrio*, and lower relative abundance of *Roseburia*, compared with controls. Among functional pathways, those for amino acid, carbohydrate, and lipid metabolism were markedly different between the two groups. Ramasamy et al. (28) showed that the abundance of *Peptococcus*, *Shimwellia*, *Propionibacterium*, *Intestinimonas*, and *Parvimonas* in knee OA was significantly higher compared with that in healthy controls. In addition, vitamin D deficiency had an effect on GMB composition, mainly reflected in *Parabacteroides*, *Butyricimonas*, and *Gordonibacter* dominating in knee OA with vitamin D deficiency, while *Peptococcus*, *Intestimonas*, *Delftia*, and *Oribacterium* were predominant in knee OA without vitamin D deficiency. Lee et al. (29) compared the GMB between RA and OA and found no difference in diversity, but the ratio of *Bacteroidetes*/*Firmicutes* (*B*/*F*) was lower in the RA group. In addition, the abundance of *Lactobacilli* and *Prevotella* was higher, while that of *Bacteroides* and *Bifidobacterium* was lower in the RA group compared with those of the OA group. Chen et al. (30) reported that the richness and diversity of GMB in OA patients were significantly reduced, and this was predominantly reflected by the decreased abundance of *Bifidobacterium longum* and *Faecalibacterium prausnitzii* and increased abundance of *Clostridium* spp. Functional prediction analysis revealed that tryptophan degradation, glutamine degradation, tyrosine degradation, propionate production, and acetate to acetyl-CoA were significantly enriched in the OA group, while triacylglycerol degradation, glycerol degradation, pentose phosphate pathway, acetyl-CoA to acetate, fructose degradation, and glycolysis were significantly enriched in the healthy control group. Boer et al. (32) reported a positive correlation between the abundance of *Streptococcus* and knee pain and synovial inflammation based on the largest cohort study to date (Rotterdam Study), which was verified in another independent cohort study (Lifelines DEEP Study). Wang et al. (35) detected decreasing diversity and richness in overweight patients with OA, with significant differences in 9 phyla and 87 genera, including *Gemmiger*, *Klebsiella*, *Akkermansia*, *Bacteroides*, *Prevotella*, *Alistipes*, and *Parabacteroides*.

### 3.4 Human studies: MR study reporting the causal relationship between GMB and OA

Yu et al. (36) firstly reported the causal relationship between GMB and OA through a two-sample MR approach based on GWAS summary statistics. *Methanobacteriaceae*, *Desulfovibrionales*, and *Ruminiclostridium 5* were identified as being negatively related to the risk of knee OA, while *Ruminococcaceae UCG003* and *Enterorhabdus* were negatively related to the risk of hip OA. In addition, 49 Gene Ontology (GO) biological processes were related to knee OA, such as regulations of melanocyte, pigment cell, and multicellular organism process. Three GO biological processes were associated with hip OA, including regulation of membrane potential, response to ammonium ion, and positive regulation of voltage-gated potassium channel activity.

### 3.5 Human studies: Clinical trials on the regulation of GMB to alleviate OA

Coulson et al. (31) observed the therapeutic effects of green-lipped mussels (GLM) and glucosamine sulfate (GS) in patients with OA. The results showed that the abundance of *Clostridium* and *Staphylococcus* decreased, while *Lactobacillus*, *Streptococcus*, and *Eubacterium* increased in the two groups. Specially, *Bifidobacterium* increased in the GLM group, while *Enterococcus* and *yeast* decreased in the GLM group, and *Bacteroides* decreased in the GS group, while *Yeasts* and *Coliforms* decreased in the GS group. At the end of the study, symptoms of OA and gastrointestinal manifestations significantly improved in both groups. *Streptococcus thermophilus* (TCI633), recently identified from human milk, is a bacterium that produces hyaluronic acid (HA) in the gastrointestinal tract. Lyu et al. (33) found that TCI633 contributed to improving serum collagen type II C-telopeptide (SCTX-II) and C-reactive protein (CRP), and alleviating OA progression. Lei et al. (34) investigated the effects of *Lactobacillus casei Shirota* (*LcS*) on patients with knee OA. In this double-blind, placebo-controlled trial, 537 patients with knee OA were randomly assigned to drink skimmed milk with *LcS* or placebo daily for 6 months. At the end of the study, patients in the *LcS* group exhibited significant improvements in Western Ontario and McMaster Universities Osteoarthritis Index (WOMAC) and visual analog scale (VAS) scores compared with those in the placebo group. C-reactive protein (CRP) levels were also significantly lower in patients receiving *LcS* compared with those in the placebo group. There was a strong linear correlation between CRP level and WOMAC and VAS scores.

### 3.6 Animal studies: Non-intervention studies reporting the association between GMB and OA

Yan et al. (37) examined the association between GMB and OA in spontaneously OA rhesus macaque and healthy rhesus macaque. GMB diversity was decreased in the OA group but the difference was not significant. The abundance of *Lactobacillus* significantly increased, while that of *Prevotella* and *Ruminococcus* decreased in the OA group. *Mollicutes*, *Tenericutes*, *Coprobacillus*, and *Faecalitalea* may be biomarkers of OA in monkeys. *F*/*B* was higher in the OA group compared with that in the control group but the difference was not statistically significant. In functional prediction analysis, 20 KEGG orthology (KO) pathways were different between the two groups, including 11 pathways that were significantly enriched in the OA group (dominated by Zinc/Manganese Transport System Permease protein and Cyclopropane-Fatty Acyl-Phospholipid synthase), and 9 pathways enriched in the healthy control group. Cintio et al. (55) compared CRP, vitamin B12, and folate in blood samples, along with the GMB, from dogs with chronic arthritis disease (AD) and healthy dogs. Compared with healthy dogs, the AD group had higher CRP and lower vitamin B12 and folate. GMB sequencing showed that the relative abundance of *Megamonas* was higher in the AD group, while those of *Paraprevotellaceae*, *Porphyromonadaceae*, and *Mogibacteriaceae* were lower. Wallace et al. (56) compared the effects of inactivity and exercise on OA and GMB in Hartley guinea pigs. The prevalence of knee OA was similar between the sedentary and exercise groups, but cartilage lesions were more severe in the sedentary group. In addition, the content of aggrecan in cartilage of the sedentary group was significantly lower than that of the exercise group. However, there were no significant differences between the two groups in cartilage thickness, collagen amount, muscle mass, subcutaneous fat, and GMB composition.

### 3.7 Animal studies: Intervention studies about GMB dysbiosis exacerbating OA progression

Huang et al. (38) found that germ-free (GF) mice without fecal transplantation had the lowest histological OA severity, while those transplanted with feces from OA patients with metabolic syndrome had the highest histological OA severity, levels of inflammatory biomarkers (IL-1β, IL-6, and MIP-1α), and intestinal mucosal permeability. High abundances of *Fusobacterium* and *Faecalibacterium* and a low abundance of *Ruminococcaceae* were strongly associated with OA severity and inflammatory markers. Guss et al. (40) investigated the effects of metabolic syndrome, obesity, and GMB on OA induced by cyclic compressive load. The study found that after 6 weeks of loading, the high-fat diet group had the most severe cartilage damage. Toll-like receptor-5 deficient (TLR5KO) mice with metabolic syndrome had cartilage damage similar to WT mice, which was alleviated by antibiotics. Collins et al. (41) found that the Modified Mankin score of high-fat diet mice was significantly higher than that of normal diet mice, and the serum lipopolysaccharide (LPS) was also significantly increased. Analysis of GMB showed a strong correlation between the abundance of *Lactobacillus* spp. and *Methanobrevibacter* spp. and Modified Mankin score. Ulici et al. (21) reported that GF mice had lower cartilage damage compared with specific-pathogen free (SPF) mice, especially among younger mice. Simultaneously, the osteophyte and serum LPS binding protein (LBP) levels of the younger GF mice were significantly lower compared with those of the SPF mice, but no significant difference in synovitis score and serum LPS was found. In addition, GMB composition was influenced by the severity of cartilage damage among SPF mice. Collins et al. (46) found that a high-fat diet (HFD) significantly increased LPS levels in synovial fluid and reduced the *B*/*F*. Nine new significant associations were also identified between GMB and OA severity. Guan et al. (48) used antibiotics to disturb GMB in mice of different genders and conducted destabilization of the medial meniscus (DMM) to establish a mouse model of OA. They found that the Osteoarthritis Research Society International (OARSI) score of female and male mice was significantly decreased after antibiotic treatment, as well as the serum levels of IL-6, TNF-α, calcium ion, and MMP-13. Luna et al. (49) performed cyclic loading to induce OA in mice and investigated the effects of HFD-induced obesity on cartilage damage and GMB. The study did not find an effect of obesity on cartilage damage, only a significant change in GMB composition was observed.

### 3.8 Animal studies: Intervention studies about regulating GMB to alleviate OA

Schott et al. (39) found that *B*/*F* and the abundance of *Actinobacteria* decreased, while the proinflammatory bacteria, dominated by *Peptostreptococcaceae* and *Peptococcaceae*, significantly increased in obese mice. Oligofructose supplementation could reverse obesity-induced GMB changes, reduce IL-12 and MCP-1, and increase IL-10. Moreover, oligofructose could also inhibit macrophage migration to the synovium and exert a chondroprotective effect by reducing the expression of Runx2, Mmp13, and COL X. Rios et al. (42) found that knee joint damage caused by a HFD was closely related to metabolic changes in mice, while prebiotics, a fiber diet, and aerobic exercise could alleviate the severity of cartilage damage. In addition, prebiotics and fiber supplementation could prevent the increase of LPS and ameliorate GMB dysbiosis in HFD-induced mice. So et al. (2010) fed mice with *Lactobacillus casei* and found that this treatment effectively improved pain, cartilage destruction, and lymphocytic infiltration, and reduced the proinflammatory cytokines (IL-1β, IL-2, IL-6, IL-12, IL17, TNF-α, IFN-γ) and MMPs (MMP1, MMP3, and MMP13). Tyndallized *Clostridium butyricum* (ID-CBT5101) could also inhibit inflammatory and bone metabolic markers (COX-2, IL-6, LTB4, COMP, MMP-2, MMP-3, MMP-9, MMP-13, TIMP-1, and TIMP-2), exerting a protective effect on cartilage (44). Kwon et al. (45) reported a therapeutic effect of probiotic complex, rosavin, and zinc on OA, mainly reflected in reductions in proinflammatory cytokines and catabolic factors accompanied by increases in anti-inflammatory cytokines and anabolic factors. Li et al. (47) reported that exercise could improve GMB diversity decreases, inflammation, and cartilage damage induced by HFD. Feeding with *Lactobacillus paracasei* subsp. *paracasei* M5 (M5) and chondroitin sulfate (CS) effectively prevented cartilage destruction and type II collagen degradation, and reduced the serum and sub-patellar fat pad leptin caused by HFD (50). Song et al. also found that there was a strong predictive relationship between *Ruminococcaceae_UCG-014*, *Streptococcus*, *Bacteroides*, and OA. Chen et al. (51) investigated the effect of moxibustion on knee cartilage injury and inflammation induced by monosodium iodoacetate (MIA). The moxibustion treatment significantly improved cartilage injury and reduced inflammatory factors in serum and cartilage. In addition, moxibustion had a regulatory effect on GMB composition. *Lactobacillus rhamnosus*, *Streptococcus thermophilus* (TCI633), and *Bifidobacterium longum* CBi0703 were all proved to exert a protective effect on cartilage through reducing inflammatory cytokines and catabolic factors in OA models (52–54); however, none of these studies elaborated on the changes of GMB.

## 4 Discussion

The causal relationship between OA and GMB has not yet been identified owing to the lack of corresponding research. Though Yu et al. (36) found a causal relationship between several microbiota and OA in a two-sample MR study based on the GWAS database, this conclusion was not supported by direct evidence. The consistent view among the studies is that the association between OA and GMB may be related to systemic or local LPS and subsequent elevation of other proinflammatory factors, such as TNF-a, IL-1b, IL-6, and CRP. These components activate the immune system and induce an inflammatory cascade. In the studies included in this systematic review, the human subjects have different geographical and ethnic backgrounds, and the experimental animals also have different genetic backgrounds. These variables can significantly affect GMB composition, leading to significant differences in the consistency of the studies. In addition, animal models of OA often use changes in diet or surgery to induce joint instability because it is difficult to develop OA spontaneously. This indicates that there is no accurate correspondence between humans and animal models, and there are even differences among various animal models. Considering the complexity of GMB composition and susceptibility to internal and external environments, it is challenging to identify a specific microorganism directly associated with OA.

It is well known that obesity and metabolic syndrome are risk factors for OA in addition to negatively affecting the GMB. According to animal studies, GMB changes have the potential to lower systemic inflammation and preserve cartilage damage in OA models. There is evidence that prebiotics or probiotics have an immunomodulatory effect that can reduce systemic inflammation and cartilage damage, as well as changes associated with OA in animals. There are limited human studies on this topic. Observational studies showed that blood LPS and LBP levels were associated with macrophage activation in the knee synovium and the severity of OA. Pain symptoms were associated with loss of GMB diversity and abundance of Streptococcus (32). According to these findings, blood toxemia or differences in GMB may contribute to OA pathogenesis. Supplementation with LcS, GLM and GS was found to alleviate OA symptoms in small clinical trials (31, 34). Nonetheless, these trials lack standardized formulations, have small sample sizes, inconsistent results, and the mechanisms mediating these outcomes remain unclear. It is necessary to conduct larger and more carefully planned randomized controlled trials before making any conclusions about the efficacy of such supplements.

Joints are traditionally considered sterile, but in recent years there have been studies that found bacterial DNA in articular cartilage and synovium. Zhao et al. (57) performed 16S rRNA gene sequencing to evaluate bacterial DNA in 16 synovial and 42 synovial fluid samples from 58 patients with OA. There were abundant and diverse bacterial DNA in these clinical samples, with *Porphyromonas* and *Bacteroides* prevalent in all 58 patients. The synovial tissue of patie

nts with OA was mainly composed of *Bacteroides*, *Megacoccus*, *Haemophilus*, *Porphyromonas*, and *Streptococcus*, while the synovial fluid was predominantly composed of *Bacteroides*. This study confirmed the presence of bacterial DNA in synovial and synovial fluid samples from OA lesions and revealed a potential correlation between bacterial DNA and lesion severity. Dunn and colleagues (58) found significant differences in the bacterial species present in human hip and knee joints. Cartilage was obtained under aseptic conditions from patients with primary knee OA and hip OA who underwent joint replacement, and from 30 fresh cadavers of similar sex, age, and body mass index (BMI) without OA. Deep sequencing of all samples showed that there were 63 different bacterial flora branches between the OA group and the control group. The OA group was dominated by *Proteus*, while the control group was dominated by *Actinomycetes* and *Clostridium*. Similarly, there were 46 branch differences in the composition of bacteria between knee and hip joints, among which hip OA was dominated by *Proteus*, while knee OA was dominated by *Actinomycetes*.

Although bacterial DNA has been found in cartilage specimens of humans and animals, no study has accurately identified where this bacterial DNA is stored: in the subchondral bone marrow, deep cartilage blood vessels, cartilage matrix, or chondrocytes. Furthermore, the process or mechanism by which these bacterial DNA or viable bacteria reach the cartilage remains unclear (58). Articular cartilage has no vessels, nerves, or lymphatic vessels, thus most of its nutrients are supplied from synovial fluid and subchondral bone marrow. The microstructure of cartilage matrix limits substance transport through pore size, charge, and molecular configuration (59). The average pore size of extracellular matrix (ECM) is estimated to be approximately 6.0 nm, whereas most bacteria have diameters ranging from 100 to 4000 nm, and bacterial spores have diameters of approximately 100 nm (60). Consequently, bacteria cannot penetrate the surface of cartilage and enter chondrocytes unless the surface is damaged by erosion. The same is true of bacterial DNA. In addition, bacterial DNA is negatively charged, and only positively charged substances can accumulate in the cartilage matrix; even neutral substances cannot enter chondrocytes (61). This phenomenon applies to most bacteria, since both Gram-positive and Gram-negative bacteria have negatively charged cell walls. In contrast, calcified cartilage, due to its increased permeability, allows passage of small solute molecules (62). During OA, there are more blood vessels and thickened tube diameters in calcified cartilage zones, which allows commensal bacterial components of the blood to transfer to calcified cartilage and subchondral bone, and even to enter normal chondrocytes through tiny cracks in the tide mark (63, 64) (**Figure 3**). Microorganisms from the gut may be stored in the subchondral bone for a long time and remain in a latent state, because the microbiota in the subchondral bone can also undergo anaerobic metabolism, which is consistent with the situation in the gut (65). Concurrently, commensal bacteria in the gut have antianabolic effects, which may inhibit osteoblasts and promote osteoclast formation through local interference with insulin-like growth factor-1 signaling, leading to bone loss (66). This transient invasion by microbiota can occur before birth; for example, *Proteus*, the predominant bacteria found in the blood and cartilage of healthy people, is part of the microbiota in the normal placenta (67).

**Figure 4:**
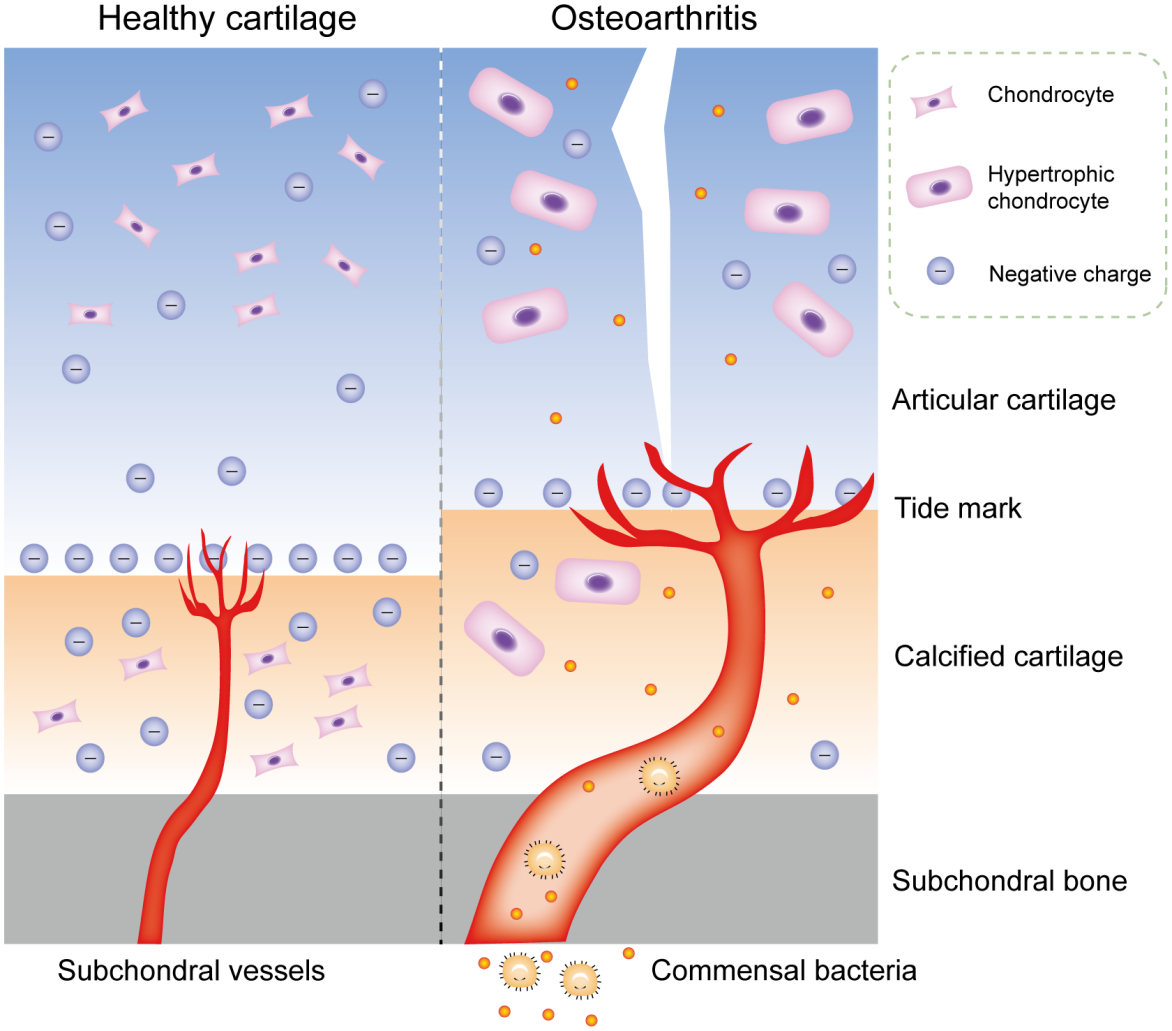
Commensal bacterial components may transfer to cartilage in OA. Physiologically, the microstructure of cartilage matrix limits substance transport through pore size, charge, and molecular configuration. During OA, there are more blood vessels and thickened tube diameters in calcified cartilage zones, which allows commensal bacterial components of the blood to transfer to calcified cartilage and subchondral bone, and even to enter normal chondrocytes through tiny cracks in the tide mark. OA, osteoarthritis.

The normal intestinal barrier acts as a mechanical barrier, chemical barrier, immune barrier, and biological barrier. Once barrier function is impaired, intestinal contents including GMB and its metabolites and immune cells will leak into the circulatory system, followed by chronic inflammation (68). Based on evidence of microbial components in the gut, blood, and joints, Tsai et al. (69) hypothesized that a “leaky” gut allows microbial-associated molecular patterns (MAMPs) correlated with dysregulation of immune signaling and promotion of inflammation to migrate from the gut to the joints, leading to OA. GMB dysbiosis can disrupt the structure and function of the intestinal barrier, and increase the porosity through several mechanisms (70). Outer membrane vesicles (OMVs), produced by pathogenic bacteria and commensal Gram-negative bacteria during the regulation of bacterial membrane proteins, have diameters ranging from 20 nm to 250 nm (71). Based on the *in vitro* activity of OMVs, Kaparakis-Liaskos and Ferrero (72) indicated that pathogens may use OMVs to disrupt the integrity of the mucosal epithelium and allow MAMPs to enter the submucosa. MAMPs could directly interact with various immune cells, including neutrophils, macrophages, and dendritic cells, contributing to pathological changes in the intestinal wall (**Figure 4**).

**Figure 5:**
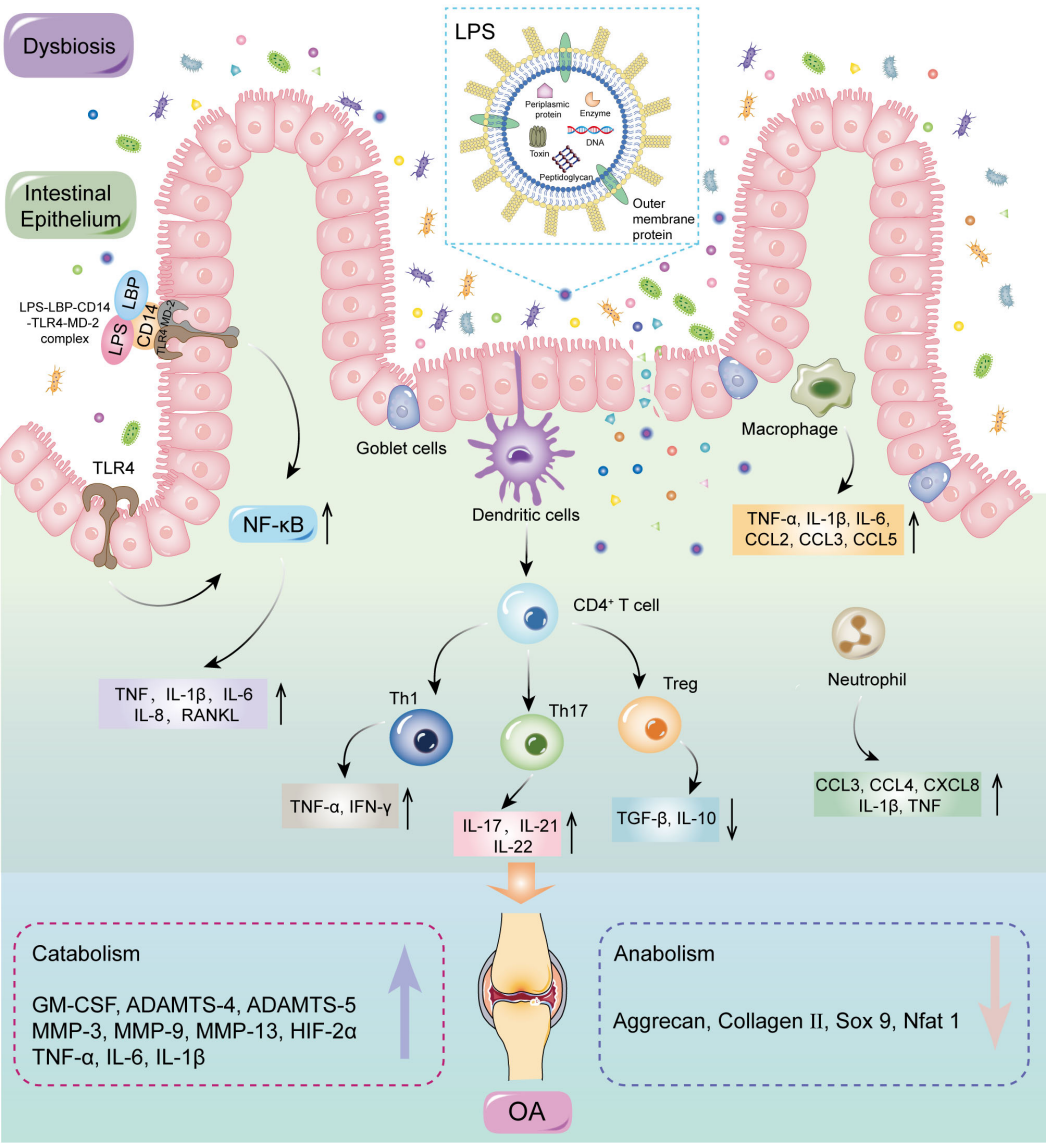
MAMPs may induce inflammation by activating the immune system. GMB dysbiosis can disrupt the structure and function of intestinal barrier, therefore allowing MAMPs to enter the submucosa. MAMPs could directly interact with various immune cells, including neutrophils, macrophages, and dendritic cells. Dendritic cells can induce the initiation and differentiation of naive CD4^+^T cells into effector cells. Activation of the inflammatory response can promote catabolism, reduce anabolism, and enhance NF-κB activation, culminating in secondary inflammation of the joints. LPS, lipopolysaccharide; LBP, LPS binding protein; CD 14, cluster of differentiation 14; TLRs, Toll-like receptors; MD-2, Myeloid differentiation protein-2; MAMPs, microbial-associated molecular patterns; NF-κB, nuclear factor kappa-B; TNF, tumor necrosis factor; IL, interleukin; CCL, CC-chemokine ligand; IFN, interferon; CXCL, CXC-chemokine ligand; GM-CSF, granulocyte–macrophage colony-stimulating factor; TGF, transforming growth factor; Th, T helper; Treg, regulatory T; MMP, matrix metallopeptidase; HIF, hypoxia inducible factor; SOX 9, SRY-Box transcription factor 9; Nfat, nuclear factor of activated T cells.

MAMPs can affect systemic and local inflammation by activating the immune system, which could accelerate OA progression (58, 73–75). The inflammation cascade in the host is predominantly dependent on pattern recognition receptors, especially Toll-like receptors (TLRs) (76, 77). MAMPs are the most common ligands for these receptors and include LPS, peptidoglycan, flagellin, and bacterial DNA, which could be carried by OMVs through the mucosal epithelium into submucosa, triggering a proinflammatory response in resident immune cells. The inflammatory factors are delivered to the joints to stimulate innate immune receptors in cartilage and synovium, thereby inducing a cascade of inflammatory reactions (78–80). LPS activates an immune response by binding to the CD14-LBP-TLR4-MD-2, resulting in increased levels of activated NF-κB, followed by the release of inflammatory factors such as TNF-α, IL-1β, IL-6, RANKL, and IL-8 (81). Activation of the inflammatory response can promote catabolism, reduce anabolism, and enhance NF-κB activation, culminating in secondary inflammation of the joints (82, 83). Dendritic cells are key antigen presenting cells mediating innate and adaptive immune responses, which can induce the initiation and differentiation of naive CD4^+^T cells into effector cells (84). In addition, Li et al. (85) elucidated the role of T cells in OA, especially T helper (Th) cells such as Th1, Th17, and regulatory T cells (Treg). T cells are clearly present in OA and produce catabolic cytokines that stimulate proteases to disrupt cartilage matrix, which accelerates OA progression by modulating the adaptive immune system (86). GMB dysbiosis determines the differentiation of primitive CD4+ T cells into effector T cells or Treg cells. The balance between Treg cells and effector T cells is critical for maintaining immune homeostasis; disorder of this balance could lead to systemic or local inflammation, including in the joints (87) (**Figure 5**).

## 5 Conclusions and future perspectives

The development of high-throughput sequencing technology and metagenomic analysis has facilitated the accurate study of GMB. In this review, we systematically summarized the current evidence supporting the “Gut-Joint” axis hypothesis. However, owing to limitations in the quantity and quality of studies, the causal role of GMB in OA has not yet been concluded. Furthermore, identification of specific microbiome (s) closely related to OA progression is challenging. Nevertheless, combined with the relevant human and animal studies, it can be concluded that there is a correlation between GMB dysbiosis and OA progression, which may be related to activation of the immune response and subsequent induction of inflammation.

There is a need for more prospective, cohort studies to determine the exact causal connections between GMB dysbiosis and OA progression. It should be noted that this low degree systemic inflammation caused by GMB dysbiosis is not unique to OA, but also exists in other diseases, such as metabolic syndrome and cardiovascular disease. In this context, we speculate that GMB may have an effect on different OA subtypes. The development of multi-omics, such as metabolomics and transcriptomics, helps to link specific metabolites, genes or signaling pathways that contribute to the regulation of GMB. In addition, single-cell sequencing is expected to reveal the correlation between different cell subsets and GMB.

## Author contributions

Conceptualization, SL and GL. Methodology, HX. Software, QW. Validation, SL, GL and HX. Formal analysis, YW. Figures, QY. Tables, AX. Writing—original draft preparation, SL and GL. Writing—review and editing, HX and FY. Supervision, JW and HZ. Project administration, JW. Funding acquisition, HZ. All authors contributed to the article and approved the submitted version.
