# Supplementary material for: “Cross-talk” between gut microbiome dysbiosis and osteoarthritis progression: a systematic review
Source: Front Immunol. 2023 Apr 25;14:1150572. doi: 10.3389/fimmu.2023.1150572 (PMC10167637; doi:10.3389/fimmu.2023.1150572)
Supplement: Supplementary file 1 [file DataSheet_1.docx]

**Supplementary Table 1.** Studies excluded after full-text assessment.

| Study | Country | Exclusion Criteria |
| --- | --- | --- |
| Aa et al. | China | RA and GMB, not for OA. |
| Armour et al. 2019 | United States | Not associated with OA. |
| Andréasson et al. 2016 | Sweden | RA and GMB, not for OA. |
| Antonenko et al. 2014 | Ukraine | No evaluation in the severity of OA. |
| Bai et al. 2021 | China | RA and GMB, not for OA. |
| Balakrishnan et al. 2021 | United States | RA and GMB, not for OA. |
| Bang et al. 2019 | Korea | No evaluation in GMB composition and OA. |
| Bellone et al. 2020 | Italy | Review and not associated with OA. |
| Benech et al. 2022 | France | FMT with inflammatory diseases, not for OA. |
| Berntson et al. 2021 | Sweden | Only for JIA, not for OA. |
| Ben-Amram et al. 2017 | Israel | RA and GMB, not for OA. |
| Block et al. 2016 | Chicago | Autoimmune arthritis, not for OA. |
| Choi et al. 2018 | Korea | Review |
| Courties et al. 2019 | France | Review |
| Donovan et al. 2018 | United States | No evaluation in GMB composition. |
| Doonan et al. 2019 | United Kingdom | RA and GMB, not for OA. |
| Edwards et al. 2021 | United States | RA and GMB, not for OA. |
| Evans et al. 2018 | United States | Inflammatory arthritis, not for OA. |
| Fan et al. 2020 | China | RA and GMB, not for OA. |
| Forbes et al. 2018 | Canada | Not associated with OA. |
| Friščić et al. 2021 | Germany | Not associated with OA and GMB |
| Gominak 2016 | United States | Not associated with OA. |
| Griffin et al. 2020 | United States | No evaluation in GMB composition. |
| Grinnell et al. 2020 | Nebraska | No evaluation in GMB composition and OA. |
| Guo et al. 2019 | China | Not associated with OA. |
| Hablot et al. 2017 | France | RA and GMB, not for OA. |
| Hahn et al. 2021 | United States | No evaluation in GMB composition |
| Haller 2010 | Germany | No evaluation in GMB composition and OA. |
| Hamamoto et al. 2020 | Japan | RA and GMB, not for OA. |
| Ilesanmi et al. 2021 | New Zealand | Osteoporosis and GMB, not for OA. |
| Jin et al. 2019 | China | Inflammation and GMB, not for OA. |
| Keirns et al. 2020 | United States | Review and not associated with OA. |
| Kell et al. 2018 | United Kingdom | Review and not associated with OA. |
| Lan et al. 2021 | China | No evaluation in the severity of OA |
| Langan et al. 2021 | United States | Autoimmune arthritis, not for OA. |
| Larsen et al. 2017 | Denmark | Review and not match the project. |
| Lázár et al. 2019 | Hungary | Not match the project. |
| Lee et al. 2019 | Korea | No evaluation in the severity of OA. |
| Li et al. 2021 | China | RA and GMB, not for OA. |
| Li et al. 2019 | China | GMB and oxidative stress, not for OA. |
| Liu et al. 2019 | China | Review |
| Lorenzo et al. 2019 | Italy | Review |
| Luu et al. 2019 | Germany | Review |
| Mendez et al. 2020 | United States | No evaluation in GMB composition. |
| Mendez et al. 2020 | United States | Not match the project. |
| Nemoto et al. 2020 | Japan | RA and GMB, not for OA. |
| Paiva et al. 2020 | Brazil | Review and not associated with OA. |
| Park et al. 2020 | Korea | Immunity and GMB, not associated with OA. |
| Pedersini et al. 2021 | Italy | No evaluation in GMB composition |
| Ponsuksili et al. 2020 | Germany | Not associated with OA. |
| Relling et al. 2018 | Germany | Metabolism and inflammation, not match. |
| Rios et al. 2021 | Canada | No evaluation in GMB composition. |
| Rogier et al. 2017 | Netherlands | Autoimmune arthritis, not for OA. |
| Rui et al. 2022 | China | RA and GMB, not for OA. |
| Rushing et al. 2022 | United States | Metabolism and OA, not for GMB. |
| Salem et al. 2019 | France | Not associated with OA. |
| Sato et al. 2017 | Japan | RA and GMB, not for OA. |
| Shang et al. 2016 | China | Not evaluate the severity of OA. |
| Sheng et al. 2022 | China | Inflammation and GMB, not for OA. |
| Shi et al. 2021 | China | RA and GMB, not for OA. |
| Stoll et al. 2019 | United States | Not associated with OA. |
| Sun et al. 2019 | China | RA and GMB, not for OA. |
| Tajik et al. 2020 | Germany | No evaluation in the severity of OA. |
| Teng et al. 2016 | United States | Autoimmune arthritis, not for OA. |
| Verma et al. 2020 | India | Reactive Arthritis, not for OA. |
| Vernocchi et al. 2020 | Italy | Only for JIA, not for OA. |
| Wang et al. 2021 | China | RA and GMB, not for OA. |
| Wang et al. 2020 | China | RA and GMB, not for OA. |
| Wang et al. 2021 | China | Aging and GMB, not for OA. |
| Wei et al. 2022 | China | Not associated with OA. |
| Wu et al. 2010 | United States | Autoimmune arthritis, not for OA. |
| Xu et al. 2020 | China | RA and GMB, not for OA. |
| Yanagisawa et al. 2018 | Japan | Inflammation and GMB, not for OA. |
| Yegorov et al. 2020 | Kazakhstan | Not associated with OA. |
| Zhang et al. 2021 | China | Aging and GMB, not for OA. |
| Zhang et al. 2020 | United States | RA and GMB, not for OA. |
| Zhou et al. 2018 | China | GMB and inflammation, not for OA. |
| Zouali 2021 | China | B lymphocytes and GMB, not for OA. |

RA, rheumatoid arthritis; OA, osteoarthritis; GMB, gut microbiome; FMT, fecal microbiota transplantation; JIA, juvenile idiopathic arthritis.

**References**

Aa LX, Fei F, Qi Q, Sun RB, Gu SH, Di ZZ, Aa JY, Wang GJ, Liu CX. Rebalancing of the gut flora and microbial metabolism is responsible for the anti-arthritis effect of kaempferol. Acta Pharmacol Sin. 2020 Jan;41(1):73-81. doi: 10.1038/s41401-019-0279-8.

Andréasson K, Alrawi Z, Persson A, Jönsson G, Marsal J. Intestinal dysbiosis is common in systemic sclerosis and associated with gastrointestinal and extraintestinal features of disease. Arthritis Res Ther. 2016 Nov 29;18(1):278. doi: 10.1186/s13075-016-1182-z.

Antonenko, A. V. and T. V. Beregova (2014). "The improvement of treatment efficacy of gastropathy associated with the use of nonsteroidal anti-inflammatory drugs in Helicobacter pylori-negative patients with osteoarthritis." Current Issues in Pharmacy and Medical Sciences 27(4): 237-239.

Armour CR, Nayfach S, Pollard KS, Sharpton TJ. A Metagenomic Meta-analysis Reveals Functional Signatures of Health and Disease in the Human Gut Microbiome. mSystems. 2019 May 14;4(4):e00332-18. doi: 10.1128/mSystems.00332-18.

Bai Y, Li Y, Marion T, Tong Y, Zaiss MM, Tang Z, Zhang Q, Liu Y, Luo Y. Resistant starch intake alleviates collagen-induced arthritis in mice by modulating gut microbiota and promoting concomitant propionate production. J Autoimmun. 2021 Jan;116:102564. doi: 10.1016/j.jaut.2020.102564.

Balakrishnan B, Luckey D, Bodhke R, Chen J, Marietta E, Jeraldo P, Murray J, Taneja V. Prevotella histicola Protects From Arthritis by Expansion of Allobaculum and Augmenting Butyrate Production in Humanized Mice. Front Immunol. 2021 May 4;12:609644. doi: 10.3389/fimmu.2021.609644.

Bang S, Yoo D, Kim SJ, Jhang S, Cho S, Kim H. Establishment and evaluation of prediction model for multiple disease classification based on gut microbial data. Sci Rep. 2019 Jul 15;9(1):10189. doi: 10.1038/s41598-019-46249-x.

Bellone M, Brevi A, Huber S. Microbiota-Propelled T Helper 17 Cells in Inflammatory Diseases and Cancer. Microbiol Mol Biol Rev. 2020 Mar 4;84(2):e00064-19. doi: 10.1128/MMBR.00064-19.

Ben-Amram H, Bashi T, Werbner N, Neuman H, Fridkin M, Blank M, Shoenfeld Y, Koren O. Tuftsin-Phosphorylcholine Maintains Normal Gut Microbiota in Collagen Induced Arthritic Mice. Front Microbiol. 2017 Jul 10;8:1222. doi: 10.3389/fmicb.2017.01222.

Benech N, Legendre P, Radoszycki L, Varriale P, Sokol H. Patient knowledge of gut microbiota and acceptability of fecal microbiota transplantation in various diseases. Neurogastroenterol Motil. 2022 Aug;34(8):e14320. doi: 10.1111/nmo.14320.

Berntson L, Hedlund-Treutiger I, Alving K. Anti-inflammatory effect of exclusive enteral nutrition in patients with juvenile idiopathic arthritis. Clin Exp Rheumatol. 2016 Sep-Oct;34(5):941-945.

Block KE, Zheng Z, Dent AL, Kee BL, Huang H. Gut Microbiota Regulates K/BxN Autoimmune Arthritis through Follicular Helper T but Not Th17 Cells. J Immunol. 2016 Feb 15;196(4):1550-7. doi: 10.4049/jimmunol.1501904.

Choi J, Hur TY, Hong Y. Influence of Altered Gut Microbiota Composition on Aging and Aging-Related Diseases. J Lifestyle Med. 2018 Jan;8(1):1-7. doi: 10.15280/jlm.2018.8.1.1.

Courties A, Berenbaum F, Sellam J. The Phenotypic Approach to Osteoarthritis: A Look at Metabolic Syndrome-Associated Osteoarthritis. Joint Bone Spine. 2019 Nov;86(6):725-730. doi: 10.1016/j.jbspin.2018.12.005.

Donovan EL, Lopes EBP, Batushansky A, Kinter M, Griffin TM. Independent effects of dietary fat and sucrose content on chondrocyte metabolism and osteoarthritis pathology in mice. Dis Model Mech. 2018 Aug 31;11(9):dmm034827. doi: 10.1242/dmm.034827.

Doonan J, Tarafdar A, Pineda MA, Lumb FE, Crowe J, Khan AM, Hoskisson PA, Harnett MM, Harnett W. The parasitic worm product ES-62 normalises the gut microbiota bone marrow axis in inflammatory arthritis. Nat Commun. 2019 Apr 5;10(1):1554. doi: 10.1038/s41467-019-09361-0.

Edwards V, Smith DL, Meylan F, Tiffany L, Poncet S, Wu WW, Phue JN, Santana-Quintero L, Clouse KA, Gabay O. Analyzing the Role of Gut Microbiota on the Onset of Autoimmune Diseases Using TNFΔARE Murine Model. Microorganisms. 2021 Dec 30;10(1):73. doi: 10.3390/microorganisms10010073.

Evans-Marin H, Rogier R, Koralov SB, Manasson J, Roeleveld D, van der Kraan PM, Scher JU, Koenders MI, Abdollahi-Roodsaz S. Microbiota-Dependent Involvement of Th17 Cells in Murine Models of Inflammatory Arthritis. Arthritis Rheumatol. 2018 Dec;70(12):1971-1983. doi: 10.1002/art.40657.

Fan Z, Yang B, Ross RP, Stanton C, Zhao J, Zhang H, Chen W. The prophylactic effects of different Lactobacilli on collagen-induced arthritis in rats. Food Funct. 2020 Apr 1;11(4):3681-3694. doi: 10.1039/c9fo02556a.

Friščić J, Dürholz K, Chen X, Engdahl C, Möller L, Schett G, Zaiss MM, Hoffmann MH. Dietary Derived Propionate Regulates Pathogenic Fibroblast Function and Ameliorates Experimental Arthritis and Inflammatory Tissue Priming. Nutrients. 2021 May 13;13(5):1643. doi: 10.3390/nu13051643.

Forbes JD, Chen CY, Knox NC, Marrie RA, El-Gabalawy H, de Kievit T, Alfa M, Bernstein CN, Van Domselaar G. A comparative study of the gut microbiota in immune-mediated inflammatory diseases-does a common dysbiosis exist? Microbiome. 2018 Dec 13;6(1):221. doi: 10.1186/s40168-018-0603-4.

Gominak SC. Vitamin D deficiency changes the intestinal microbiome reducing B vitamin production in the gut. The resulting lack of pantothenic acid adversely affects the immune system, producing a "pro-inflammatory" state associated with atherosclerosis and autoimmunity. Med Hypotheses. 2016 Sep;94:103-7. doi: 10.1016/j.mehy.2016.07.007.

Griffin TM, Batushansky A, Hudson J, Lopes EBP. Correlation network analysis shows divergent effects of a long-term, high-fat diet and exercise on early stage osteoarthritis phenotypes in mice. J Sport Health Sci. 2020 Mar;9(2):119-131. doi: 10.1016/j.jshs.2019.05.008.

Grinnell M, Ogdie A, Wipfler K, Michaud K. Probiotic Use and Psoriatic Arthritis Disease Activity. ACR Open Rheumatol. 2020 Jun;2(6):330-334. doi: 10.1002/acr2.11143.

Guo LX, Wang HY, Liu XD, Zheng JY, Tang Q, Wang XN, Liu JQ, Yin HQ, Miao B, Liang YL, Liu LF, Xin GZ. Saponins from Clematis mandshurica Rupr. regulates gut microbiota and its metabolites during alleviation of collagen-induced arthritis in rats. Pharmacol Res. 2019 Nov;149:104459. doi: 10.1016/j.phrs.2019.104459.

Hablot J, Peyrin-Biroulet L, Kokten T, El Omar R, Netter P, Bastien C, Jouzeau JY, Sokol H, Moulin D. Experimental colitis delays and reduces the severity of collagen-induced arthritis in mice. PLoS One. 2017 Sep 19;12(9):e0184624. doi: 10.1371/journal.pone.0184624.

Hahn AK, Wallace CW, Welhaven HD, Brooks E, McAlpine M, Christiansen BA, Walk ST, June RK. The microbiome mediates epiphyseal bone loss and metabolomic changes after acute joint trauma in mice. Osteoarthritis Cartilage. 2021 Jun;29(6):882-893. doi: 10.1016/j.joca.2021.01.012.

Haller D. Nutrigenomics and IBD: the intestinal microbiota at the cross-road between inflammation and metabolism. J Clin Gastroenterol. 2010 Sep;44 Suppl 1:S6-9. doi: 10.1097/MCG.0b013e3181dd8b76.

Hamamoto Y, Ouhara K, Munenaga S, Shoji M, Ozawa T, Hisatsune J, Kado I, Kajiya M, Matsuda S, Kawai T, Mizuno N, Fujita T, Hirata S, Tanimoto K, Nakayama K, Kishi H, Sugiyama E, Kurihara H. Effect of Porphyromonas gingivalis infection on gut dysbiosis and resultant arthritis exacerbation in mouse model. Arthritis Res Ther. 2020 Oct 19;22(1):249. doi: 10.1186/s13075-020-02348-z.

Ilesanmi-Oyelere BL, Roy NC, Kruger MC. Modulation of Bone and Joint Biomarkers, Gut Microbiota, and Inflammation Status by Synbiotic Supplementation and Weight-Bearing Exercise: Human Study Protocol for a Randomized Controlled Trial. JMIR Res Protoc. 2021 Oct 26;10(10):e30131. doi: 10.2196/30131.

Jin DX, He JF, Zhang KQ, Luo XG, Zhang TC. EtOAc extract of H. attenuatum Choisy inhibits inflammation by suppressing the NF-κB and MAPK pathways and modulating the gut microbiota. Phytomedicine. 2019 Apr;57:292-304. doi: 10.1016/j.phymed.2018.12.037.

Keirns BH, Lucas EA, Smith BJ. Phytochemicals affect T helper 17 and T regulatory cells and gut integrity: implications on the gut-bone axis. Nutr Res. 2020 Nov;83:30-48. doi: 10.1016/j.nutres.2020.08.006.

Kell DB, Pretorius E. No effects without causes: the Iron Dysregulation and Dormant Microbes hypothesis for chronic, inflammatory diseases. Biol Rev Camb Philos Soc. 2018 Aug;93(3):1518-1557. doi: 10.1111/brv.12407.

Lan H, Hong W, Qian D, Peng F, Li H, Liang C, Du M, Gu J, Mai J, Bai B, Peng G. Quercetin modulates the gut microbiota as well as the metabolome in a rat model of osteoarthritis. Bioengineered. 2021 Dec;12(1):6240-6250. doi: 10.1080/21655979.2021.1969194.

Langan D, Perkins DJ, Vogel SN, Moudgil KD. Microbiota-Derived Metabolites, Indole-3-aldehyde and Indole-3-acetic Acid, Differentially Modulate Innate Cytokines and Stromal Remodeling Processes Associated with Autoimmune Arthritis. Int J Mol Sci. 2021 Feb 18;22(4):2017. doi: 10.3390/ijms22042017.

Larsen JM. The immune response to Prevotella bacteria in chronic inflammatory disease. Immunology. 2017 Aug;151(4):363-374. doi: 10.1111/imm.12760.

Lázár B, Brenner GB, Makkos A, Balogh M, László SB, Al-Khrasani M, Hutka B, Bató E, Ostorházi E, Juhász J, Kemény Á, László T, Tiszlavicz L, Bihari Z, Giricz Z, Szabó D, Helyes Z, Ferdinandy P, Gyires K, Zádori ZS. Lack of Small Intestinal Dysbiosis Following Long-Term Selective Inhibition of Cyclooxygenase-2 by Rofecoxib in the Rat. Cells. 2019 Mar 15;8(3):251. doi: 10.3390/cells8030251.

Lee S, Koh J, Chang Y, Kim HY, Chung DH. Invariant NKT Cells Functionally Link Microbiota-Induced Butyrate Production and Joint Inflammation. J Immunol. 2019 Dec 15;203(12):3199-3208. doi: 10.4049/jimmunol.1801314.

Li B, Du P, Smith EE, Wang S, Jiao Y, Guo L, Huo G, Liu F. In vitro and in vivo evaluation of an exopolysaccharide produced by Lactobacillus helveticus KLDS1.8701 for the alleviative effect on oxidative stress. Food Funct. 2019 Mar 20;10(3):1707-1717. doi: 10.1039/c8fo01920g.

Li Y, Dai M, Wang L, Wang G. Polysaccharides and glycosides from Aralia echinocaulis protect rats from arthritis by modulating the gut microbiota composition. J Ethnopharmacol. 2021 Apr 6;269:113749. doi: 10.1016/j.jep.2020.113749.

Liu Y, Ding W, Wang HL, Dai LL, Zong WH, Wang YZ, Bi J, Han W, Dong GJ. Gut microbiota and obesity-associated osteoarthritis. Osteoarthritis Cartilage. 2019 Sep;27(9):1257-1265. doi: 10.1016/j.joca.2019.05.009.

Lorenzo D, GianVincenzo Z, Carlo Luca R, Karan G, Jorge V, Roberto M, Javad P. Oral-Gut Microbiota and Arthritis: Is There an Evidence-Based Axis? J Clin Med. 2019 Oct 22;8(10):0. doi: 10.3390/jcm8101753.

Luu M, Visekruna A. Short-chain fatty acids: Bacterial messengers modulating the immunometabolism of T cells. Eur J Immunol. 2019 Jun;49(6):842-848. doi: 10.1002/eji.201848009.

Mendez ME, Murugesh DK, Sebastian A, Hum NR, McCloy SA, Kuhn EA, Christiansen BA, Loots GG. Antibiotic Treatment Prior to Injury Improves Post-Traumatic Osteoarthritis Outcomes in Mice. Int J Mol Sci. 2020 Sep 3;21(17):6424. doi: 10.3390/ijms21176424.

Mendez ME, Sebastian A, Murugesh DK, Hum NR, McCool JL, Hsia AW, Christiansen BA, Loots GG. LPS-Induced Inflammation Prior to Injury Exacerbates the Development of Post-Traumatic Osteoarthritis in Mice. J Bone Miner Res. 2020 Nov;35(11):2229-2241. doi: 10.1002/jbmr.4117.

Nemoto N, Takeda Y, Nara H, Araki A, Gazi MY, Takakubo Y, Naganuma Y, Takagi M, Asao H. Analysis of intestinal immunity and flora in a collagen-induced mouse arthritis model: differences during arthritis progression. Int Immunol. 2020 Jan 9;32(1):49-56. doi: 10.1093/intimm/dxz058.

Paiva IHR, Duarte-Silva E, Peixoto CA. The role of prebiotics in cognition, anxiety, and depression. Eur Neuropsychopharmacol. 2020 May;34:1-18. doi: 10.1016/j.euroneuro.2020.03.006.

Park MR, Shin M, Mun D, Jeong SY, Jeong DY, Song M, Ko G, Unno T, Kim Y, Oh S. Probiotic Lactobacillus fermentum strain JDFM216 improves cognitive behavior and modulates immune response with gut microbiota. Sci Rep. 2020 Dec 10;10(1):21701. doi: 10.1038/s41598-020-77587-w.

Pedersini P, Savoldi M, Berjano P, Villafañe JH. A probiotic intervention on pain hypersensitivity and microbiota composition in patients with osteoarthritis pain: Study protocol for a randomized controlled trial. Arch Rheumatol. 2021 Jan 27;36(2):296-301. doi: 10.46497/ArchRheumatol.2021.7719.

Ponsuksili S, Reyer H, Hadlich F, Weber F, Trakooljul N, Oster M, Siengdee P, Muráni E, Rodehutscord M, Camarinha-Silva A, Bennewitz J, Wimmers K. Identification of the Key Molecular Drivers of Phosphorus Utilization Based on Host miRNA-mRNA and Gut Microbiome Interactions. Int J Mol Sci. 2020 Apr 17;21(8):2818. doi: 10.3390/ijms21082818.

Relling I, Akcay G, Fangmann D, Knappe C, Schulte DM, Hartmann K, Müller N, Türk K, Dempfle A, Franke A, Schreiber S, Laudes M. Role of wnt5a in Metabolic Inflammation in Humans. J Clin Endocrinol Metab. 2018 Nov 1;103(11):4253-4264. doi: 10.1210/jc.2018-01007.

Rios JL, Hart DA, Reimer RA, Herzog W. Prebiotic and Exercise Do Not Alter Knee Osteoarthritis in a Rat Model of Established Obesity. Cartilage. 2021 Dec;13(2_suppl):1456S-1466S. doi: 10.1177/1947603520959399.

Rogier R, Ederveen THA, Boekhorst J, Wopereis H, Scher JU, Manasson J, Frambach SJCM, Knol J, Garssen J, van der Kraan PM, Koenders MI, van den Berg WB, van Hijum SAFT, Abdollahi-Roodsaz S. Aberrant intestinal microbiota due to IL-1 receptor antagonist deficiency promotes IL-17- and TLR4-dependent arthritis. Microbiome. 2017 Jun 23;5(1):63. doi: 10.1186/s40168-017-0278-2.

Rui Z, Zhang L, Li X, Han J, Yuan Y, Ding H, Liu Y, Ding X. Pterostilbene exert an anti-arthritic effect by attenuating inflammation, oxidative stress, and alteration of gut microbiota. J Food Biochem. 2022 May;46(5):e14011. doi: 10.1111/jfbc.14011.

Rushing BR, McRitchie S, Arbeeva L, Nelson AE, Azcarate-Peril MA, Li YY, Qian Y, Pathmasiri W, Sumner SCJ, Loeser RF. Fecal metabolomics reveals products of dysregulated proteolysis and altered microbial metabolism in obesity-related osteoarthritis. Osteoarthritis Cartilage. 2022 Jan;30(1):81-91. doi: 10.1016/j.joca.2021.10.006.

Salem F, Kindt N, Marchesi JR, Netter P, Lopez A, Kokten T, Danese S, Jouzeau JY, Peyrin-Biroulet L, Moulin D. Gut microbiome in chronic rheumatic and inflammatory bowel diseases: Similarities and differences. United European Gastroenterol J. 2019 Oct;7(8):1008-1032. doi: 10.1177/2050640619867555.

Sato K, Takahashi N, Kato T, Matsuda Y, Yokoji M, Yamada M, Nakajima T, Kondo N, Endo N, Yamamoto R, Noiri Y, Ohno H, Yamazaki K. Aggravation of collagen-induced arthritis by orally administered Porphyromonas gingivalis through modulation of the gut microbiota and gut immune system. Sci Rep. 2017 Jul 31;7(1):6955. doi: 10.1038/s41598-017-07196-7.

Shi W, Ye H, Deng Y, Chen S, Xiao W, Wang Z, Xiong Z, Zhao L. Yaobitong capsules reshape and rebalance the gut microbiota and metabolites of arthritic rats: An integrated study of microbiome and fecal metabolomics analysis. J Chromatogr B Analyt Technol Biomed Life Sci. 2022 Feb 1;1190:123096. doi: 10.1016/j.jchromb.2021.123096.

Shang Q, Yin Y, Zhu L, Li G, Yu G, Wang X. Degradation of chondroitin sulfate by the gut microbiota of Chinese individuals. Int J Biol Macromol. 2016 May;86:112-8. doi: 10.1016/j.ijbiomac.2016.01.055.

Sheng K, Yang J, Xu Y, Kong X, Wang J, Wang Y. Alleviation effects of grape seed proanthocyanidin extract on inflammation and oxidative stress in a D-galactose-induced aging mouse model by modulating the gut microbiota. Food Funct. 2022 Feb 7;13(3):1348-1359. doi: 10.1039/d1fo03396d.

Stoll ML, Pierce MK, Watkins JA, Zhang M, Weiss PF, Weiss JE, Elson CO, Cron RQ, Kumar R, Morrow CD, Schoeb TR. Akkermansia muciniphila is permissive to arthritis in the K/BxN mouse model of arthritis. Genes Immun. 2019 Feb;20(2):158-166. doi: 10.1038/s41435-018-0024-1.

Sun Y, Chen Q, Lin P, Xu R, He D, Ji W, Bian Y, Shen Y, Li Q, Liu C, Dong K, Tang YW, Pei Z, Yang L, Lu H, Guo X, Xiao L. Characteristics of Gut Microbiota in Patients With Rheumatoid Arthritis in Shanghai, China. Front Cell Infect Microbiol. 2019 Oct 23;9:369. doi: 10.3389/fcimb.2019.00369.

Tajik N, Frech M, Schulz O, Schälter F, Lucas S, Azizov V, Dürholz K, Steffen F, Omata Y, Rings A, Bertog M, Rizzo A, Iljazovic A, Basic M, Kleyer A, Culemann S, Krönke G, Luo Y, Überla K, Gaipl US, Frey B, Strowig T, Sarter K, Bischoff SC, Wirtz S, Cañete JD, Ciccia F, Schett G, Zaiss MM. Targeting zonulin and intestinal epithelial barrier function to prevent onset of arthritis. Nat Commun. 2020 Apr 24;11(1):1995. doi: 10.1038/s41467-020-15831-7.

Teng F, Klinger CN, Felix KM, Bradley CP, Wu E, Tran NL, Umesaki Y, Wu HJ. Gut Microbiota Drive Autoimmune Arthritis by Promoting Differentiation and Migration of Peyer's Patch T Follicular Helper Cells. Immunity. 2016 Apr 19;44(4):875-88. doi: 10.1016/j.immuni.2016.03.013.

Verma A, Sharda S, Rathi B, Somvanshi P, Pandey BD. Elucidating potential molecular signatures through host-microbe interactions for reactive arthritis and inflammatory bowel disease using combinatorial approach. Sci Rep. 2020 Sep 15;10(1):15131. doi: 10.1038/s41598-020-71674-8.

Vernocchi P, Marini F, Capuani G, Tomassini A, Conta G, Del Chierico F, Malattia C, De Benedetti F, Martini A, Dallapiccola B, van Dijkhuizen EHP, Miccheli A, Putignani L. Fused Omics Data Models Reveal Gut Microbiome Signatures Specific of Inactive Stage of Juvenile Idiopathic Arthritis in Pediatric Patients. Microorganisms. 2020 Oct 6;8(10):1540. doi: 10.3390/microorganisms8101540.

Wang B, He Y, Tang J, Ou Q, Lin J. Alteration of the gut microbiota in tumor necrosis factor-α antagonist-treated collagen-induced arthritis mice. Int J Rheum Dis. 2020 Apr;23(4):472-479. doi: 10.1111/1756-185X.13802.

Wang H, Ong E, Kao JY, Sun D, He Y. Reverse Microbiomics: A New Reverse Dysbiosis Analysis Strategy and Its Usage in Prediction of Autoantigens and Virulent Factors in Dysbiotic Gut Microbiomes From Rheumatoid Arthritis Patients. Front Microbiol. 2021 Feb 25;12:633732. doi: 10.3389/fmicb.2021.633732.

Wang W, Liu F, Xu C, Liu Z, Ma J, Gu L, Jiang Z, Hou J. Lactobacillus plantarum 69-2 Combined with Galacto-Oligosaccharides Alleviates d-Galactose-Induced Aging by Regulating the AMPK/SIRT1 Signaling Pathway and Gut Microbiota in Mice. J Agric Food Chem. 2021 Mar 10;69(9):2745-2757. doi: 10.1021/acs.jafc.0c06730.

Wei J, Zhang Y, Dalbeth N, Terkeltaub R, Yang T, Wang Y, Yang Z, Li J, Wu Z, Zeng C, Lei G. Association Between Gut Microbiota and Elevated Serum Urate in Two Independent Cohorts. Arthritis Rheumatol. 2022 Apr;74(4):682-691. doi: 10.1002/art.42009.

Wu HJ, Ivanov II, Darce J, Hattori K, Shima T, Umesaki Y, Littman DR, Benoist C, Mathis D. Gut-residing segmented filamentous bacteria drive autoimmune arthritis via T helper 17 cells. Immunity. 2010 Jun 25;32(6):815-27. doi: 10.1016/j.immuni.2010.06.001.

Xu H, Cao J, Li X, Lu X, Xia Y, Fan D, Zhao H, Ju D, Xiao C. Regional Differences in the Gut Microbiota and Gut-Associated Immunologic Factors in the Ileum and Cecum of Rats With Collagen-Induced Arthritis. Front Pharmacol. 2020 Nov 24;11:587534. doi: 10.3389/fphar.2020.587534.

Yanagisawa N, Ueshiba H, Abe Y, Kato H, Higuchi T, Yagi J. Outer Membrane Protein of Gut Commensal Microorganism Induces Autoantibody Production and Extra-Intestinal Gland Inflammation in Mice. Int J Mol Sci. 2018 Oct 19;19(10):3241. doi: 10.3390/ijms19103241.

Yegorov S, Babenko D, Kozhakhmetov S, Akhmaltdinova L, Kadyrova I, Nurgozhina A, Nurgaziyev M, Good SV, Hortelano GH, Yermekbayeva B, Kushugulova A. Psoriasis Is Associated With Elevated Gut IL-1α and Intestinal Microbiome Alterations. Front Immunol. 2020 Oct 1;11:571319. doi: 10.3389/fimmu.2020.571319.

Zhang L, Song P, Zhang X, Metea C, Schleisman M, Karstens L, Leung E, Zhang J, Xu Q, Liu Y, Asquith M, Chu CQ. Alpha-Glucosidase Inhibitors Alter Gut Microbiota and Ameliorate Collagen-Induced Arthritis. Front Pharmacol. 2020 Feb 4;10:1684. doi: 10.3389/fphar.2019.01684.

Zhang X, Yang Y, Su J, Zheng X, Wang C, Chen S, Liu J, Lv Y, Fan S, Zhao A, Chen T, Jia W, Wang X. Age-related compositional changes and correlations of gut microbiome, serum metabolome, and immune factor in rats. Geroscience. 2021 Apr;43(2):709-725. doi: 10.1007/s11357-020-00188-y.

Zhou Y, Ni X, Wen B, Duan L, Sun H, Yang M, Zou F, Lin Y, Liu Q, Zeng Y, Fu X, Pan K, Jing B, Wang P, Zeng D. Appropriate dose of Lactobacillus buchneri supplement improves intestinal microbiota and prevents diarrhoea in weaning Rex rabbits. Benef Microbes. 2018 Apr 25;9(3):401-416. doi: 10.3920/BM2017.0055.

Zouali M. B lymphocytes, the gastrointestinal tract and autoimmunity. Autoimmun Rev. 2021 Apr;20(4):102777. doi: 10.1016/j.autrev.2021.102777.
