# Supplementary material for: “Cross-talk” between gut microbiome dysbiosis and osteoarthritis progression: a systematic review
Source: Front Immunol. 2023 Apr 25;14:1150572. doi: 10.3389/fimmu.2023.1150572 (PMC10167637; doi:10.3389/fimmu.2023.1150572)
Supplement: Supplementary file 2 [file DataSheet_2.docx]

**Supplementary Table 2.** Basic characteristics of participants in human studies.

| Study | Country | Group | Gender (Male/  Female) | Age  (Years) | BMI (Kg/m^2^)/  Body Weight (Kg) |
| --- | --- | --- | --- | --- | --- |
| Wei et al. 2021 | China | SHOA | 25/75 | 70.9±8.3 | 24.1±3.8 |
|  |  | Control | 43/57 | 62.8±8.9 | 23.9±3.5 |
| Ramasamy  et al. 2021 | India | KOA_VDD | 1/6 | 52.0±7.2 | 28.2±2.7 |
|  |  | KOA | 1/3 | 50.0±9.7 | 27.5±2.8 |
|  |  | VDD | 7/0 | 44.0±8.1 | 28.9±2.6 |
|  |  | Normal (NVD) | 7/0 | 37.7±12.7 | 22.9±2.6 |
| Lee et al. 2019 | Korea | RA | / | 55.0±5.2 | 24.7±3.2 |
|  |  | OA | / | 59.8±8.2 | 24.5±2.9 |
| Chen et al. 2021 | United Kingdom | OA | 0/57 | 65.0±8.0 | 26.0±4.0 |
|  |  | Control | 0/57 | 62.0±8.0 | 25.0±4.0 |
| Coulson  et al. 2013 | Australia | GLM | 16/5 | 56.7±8.9 | 31.3±6.1 |
|  |  | GS | 12/17 | 60.0±8.6 | 30.2±4.8 |
| Boer et al. 2019 | Netherlands | / | 606/821 | 56.9±5.9 | 27.5±4.5 |
| Lyu et al. 2020 | China | TCI633 | 8/22 | 60.8±12.2 | 64±11.1 |
|  |  | Placebo | 6/31 | 65.1±9.3 | 62.7±11.0 |
| Lei et al. 2017 | China | LcS | 95/120 | 66.5±5.2 | 24.3±2.5 |
|  |  | Placebo | 97/121 | 67.2±4.8 | 25.1±3.1 |
| Wang et al. 2021 | China | Overweight OA | 25/61 | 62 (50-72) | / |
|  |  | Control | 40/56 | 64 (50-76) | / |

BMI, body mass index; OA, osteoarthritis; SHOA, symptomatic hand OA; VDD, vitamin D deficiency; NVD, normal vitamin D; RA, rheumatoid arthritis; GLM, green-lipped mussel; GS, glucosamine sulphate; TCI633, Streptococcus thermophilus; LcS, Lactobacillus casei Shirota.
