# Supplementary material for: “Cross-talk” between gut microbiome dysbiosis and osteoarthritis progression: a systematic review
Source: Front Immunol. 2023 Apr 25;14:1150572. doi: 10.3389/fimmu.2023.1150572 (PMC10167637; doi:10.3389/fimmu.2023.1150572)
Supplement: Supplementary file 3 [file DataSheet_3.docx]

**AHRQ Risk of Bias Evaluation**

| Study | Selection Bias | Performance Bias | Attrition Bias | Detection Bias | Reporting Bias |
| --- | --- | --- | --- | --- | --- |
| Wei et al. 2021 | Low | High | Unclear | Unclear | Unclear |
| Ramasamy et al. 2021 | Low | Unclear | Unclear | Unclear | Low |
| Lee et al. 2019 | Low | High | Unclear | High | Low |
| Chen et al. 2021 | Low | Unclear | Unclear | Low | Low |
| Coulson et al. 2013 | Low | Low | Low | Low | Low |
| Boer et al. 2019 | Low | High | Unclear | Low | Unclear |
| Lyu et al. 2020 | Low | Low | Unclear | Low | Low |
| Lei et al. 2017 | Low | Low | Unclear | Low | Low |
| Wang et al. 2021 | High | Unclear | Unclear | High | Low |

AHRQ, Agency for Healthcare Research and Quality.
