# Supplementary material for: “Cross-talk” between gut microbiome dysbiosis and osteoarthritis progression: a systematic review
Source: Front Immunol. 2023 Apr 25;14:1150572. doi: 10.3389/fimmu.2023.1150572 (PMC10167637; doi:10.3389/fimmu.2023.1150572)
Supplement: Supplementary file 4 [file DataSheet_4.docx]

**CAMARADES Quality Evaluation**

| Study | Sample Size Calculation | Random Allocation | Blinded Evaluation of Outcomes | Appropriate Animal Model | Animal Welfare | Peer review | Conflict of Interest Declaration |
| --- | --- | --- | --- | --- | --- | --- | --- |
| Yan et al. 2021 | No | No | No | Yes | Yes | No | Yes |
| Huang et al. 2020 | No | No | Yes | Yes | Yes | No | No |
| Schott et al. 2018 | No | Yes | Yes | Yes | Yes | No | No |
| Guss et al. 2020 | No | No | No | Yes | Yes | No | No |
| Collins et al. 2015 | No | No | No | Yes | Yes | No | Yes |
| Ulici et al. 2018 | No | No | Yes | Yes | Yes | No | No |
| Rios et al. 2019 | No | Yes | Yes | Yes | Yes | No | Yes |
| So et al. 2011 | No | No | No | Yes | Yes | No | Yes |
| Sim et al. 2018 | No | Yes | No | Yes | Yes | No | Yes |
| Kwon et al. 2017 | No | Yes | No | Yes | Yes | No | Yes |
| Collins et al. 2021 | No | No | No | Yes | Yes | No | Yes |
| Li et al. 2021 | No | Yes | Yes | Yes | Yes | No | Yes |
| Guan et al. 2020 | No | No | Yes | Yes | Yes | No | Yes |
| Luna et al. 2021 | No | No | No | Yes | Yes | No | No |
| Song et al. 2020 | No | Yes | Yes | Yes | Yes | No | Yes |
| Chen et al. 2020 | No | Yes | No | Yes | Yes | No | Yes |
| Jhun et al. 2021 | No | Yes | Yes | Yes | Yes | No | Yes |
| Lin et al. 2021 | No | Yes | No | Yes | Yes | No | Yes |
| Henrotin et al. 2021 | No | Yes | No | Yes | Yes | No | Yes |
| Cintio et al. 2020 | No | No | No | Yes | Yes | No | Yes |
| Wallace et al. 2019 | No | Yes | Yes | Yes | Yes | No | Yes |
